# Water‐Induced Phase Separation for Anti‐Swelling Hydrogel Adhesives in Underwater Soft Electronics

**DOI:** 10.1002/advs.202304780

**Published:** 2023-09-26

**Authors:** Min Li, Honglang Lu, Menghan Pi, Hui Zhou, Yufei Wang, Bin Yan, Wei Cui, Rong Ran

**Affiliations:** ^1^ College of Polymer Science and Engineering Sichuan University Chengdu 610065 China

**Keywords:** phase separation, hydrogel, swelling resistance, underwater adhesion, underwater electronics

## Abstract

The development of hydrogel‐based underwater electronics has gained significant attention due to their flexibility and portability compared to conventional rigid devices. However, common hydrogels face challenges such as swelling and poor underwater adhesion, limiting their practicality in water environments. Here, a water‐induced phase separation strategy to fabricate hydrogels with enhanced anti‐swelling properties and underwater adhesion is presented. By leveraging the contrasting affinity of different polymer chains to water, a phase‐separated structure with rich hydrophobic and dilute hydrophilic polymer phases is achieved. This dual‐phase structure, meticulously characterized from the macroscopic to the nanoscale, confers the hydrogel network with augmented retractive elastic forces and facilitates efficient water drainage at the gel‐substrate interface. As a result, the hydrogel exhibits remarkable swelling resistance and long‐lasting adhesion to diverse substrates. Additionally, the integration of carboxylic multiwalled carbon nanotubes into the hydrogel system preserves its anti‐swelling and adhesion properties while imparting superior conductivity. The conductive phase‐separated hydrogel exhibited great potential in diverse underwater applications, including sensing, communication, and energy harvesting. This study elucidates a facile strategy for designing anti‐swelling underwater adhesives by leveraging the ambient solvent effect, which is expected to offer some insights for the development of next‐generation adhesive soft materials tailored for aqueous environments.

## Graphical abstract

Water‐induced phase separation strategy is utilized to develop anti‐swelling hydrogels for underwater adhesion. The formation of hydrophobic domains at the gel surface facilitates water drainage at the interface, resulting in satisfactory adhesion capability of the gel underwater. By introducing carboxylic multiwalled carbon nanotubes into the hydrogel system, the hydrogel adhesive holds great potential in underwater sensing, communication, and energy harvesting.

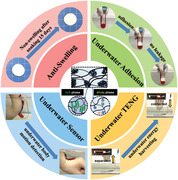

## 1 Introduction

Hydrogels, as unique soft and wet materials renowned for their high deformability and excellent biocompatibility, have witnessed extensive applications in the field of biomedical research for several decades. Notably, they have been employed in diverse areas, including wound dressing,^[^ ^1^ ^]^ drug delivery,^[^ ^2^ ^]^ and tissue adhesive.^[^ ^3^ ^]^ Possessing a distinct 3D hydrated network structure, hydrogels exhibit solid properties at a macroscopic scale while displaying liquid‐like behavior at a microscopic level. This fascinating characteristic has facilitated the facile modification of hydrogels by incorporating functional additives.^[^ ^4^ ^]^ For instance, insulating hydrogels can be transformed into exceptional conductors through the introduction of compatible ions or conductive fillers into their network.^[^ ^5^ ^]^ Consequently, their applications have expanded from traditional fields to cutting‐edge domains such as artificial skin,^[^ ^6^ ^]^ flexible displays,^[^ ^7^ ^]^ and wearable sensors.^[^ ^8^ ^]^ Nowadays, an emerging field named hydrogel electronics rapidly evolves, paving the way for the development of next‐generation soft materials for human‐machine interfaces, energy harvesting, etc.

A long‐standing challenge in the field of hydrogel electronics lies in achieving robust adhesion between hydrogels and solid substrates, particularly for ensuring the proper functioning of soft‐rigid hybrid devices. The inherent disparities between the soft, wet hydrogels and the rigid, dry substrates pose a significant hurdle to securely bonding these two antagonistic materials. This challenge becomes even more formidable in underwater settings, as hydrogels tend to swell in aqueous environments. While swelling is advantageous for certain applications like plugging agents and super absorbents, it becomes a drawback when hydrogels are employed in underwater electronics. Swelling often leads to volume expansion of hydrogels by diluting polymer chains in the network,^[^ ^9^ ^]^ diminishing the bonding sites at the gel‐substrate interface and compromising the mechanical reliability of composite devices.^[^ ^10^ ^]^ Consequently, numerous hydrogels exhibit adhesive properties in air but tend to lose their stickiness once equilibrated in water, significantly increasing the risk of interfacial failure in underwater electronics.

To address this challenge, extensive research efforts have been devoted to endowing hydrogels with satisfactory underwater adhesion capabilities, employing both physical and chemical approaches. For instance, Rao et al. designed underwater hydrogel adhesives by mimicking the surface architecture of clingfish suction discs.^[^ ^11^ ^]^ This bio‐inspired hydrogel surface facilitated water drainage at the interface, enabling efficient contact between the gel and diverse substrates for robust adhesion underwater. In a different approach, Fan et al. utilized cation‐π complex‐aided copolymerization to synthesize sequence‐controlled polymers with exceptional stickiness,^[^ ^12^ ^]^ providing novel chemical insights for developing underwater hydrogel adhesives. Additionally, other methods, such as topological entanglement,^[^ ^13^ ^]^ supramolecular chemistry,^[^ ^14^ ^]^ surface modification,^[^ ^15^ ^]^ have also proven effective in enhancing the wet adhesion capabilities of hydrogels. However, most existing methods primarily focus on the structural engineering or chemical design of the hydrogel system itself, often overlooking the potential positive effects of the ambient environment, particularly the solvent, on the underwater adhesion of hydrogels.

One promising strategy that has recently gained attention is mixed‐solvent‐induced phase separation, which can transform various non‐adhesive hydrogels into robust glues without the need for specific chemical treatment or surface engineering.^[^ ^16^ ^]^ The key mechanism lies in the contrasting affinity of polymer chains to their good and poor solvents. By subjecting a hydrogel in a mixture of their good and poor solvents, the original single‐phase structure undergoes phase separation, forming polymer‐rich and polymer‐dilute phases.^[^ ^17^ ^]^ The resulting inhomogeneous structure enhances hydrogel adhesion in two ways. First, the polymer‐rich phases at the gel surface form dense arrays of noncovalent bonds with the substrate, improving the work of adhesion and facilitating force transmission from the interfacial crack front to the bulk gel. Second, the phase‐separated structure with soft/hard regions allows for significant mechanical dissipation of the gel upon interfacial separation. Such a synergy leads to impressive adhesion energy of the phase‐separated (PS) hydrogel that rivals the best‐in‐class gel adhesives.^[^ ^18^ ^]^

One obvious drawback of the mixed‐solvent‐induced phase separation method is its strict selectivity toward solvents. Only specific combinations of good and poor solvents lead to phase separation and the resulting adhesiveness of hydrogels. Moreover, the phase separation process is reversible, causing the PS hydrogel to revert to its initial single‐phase structure when immersed in water,^[^ ^19^ ^]^ returning to a non‐adhesive material. These limitations greatly restrict the practicability of the solvent‐induced phase separation method. Therefore, the development of an innovative strategy that utilizes accessible solvents to create long‐lasting underwater hydrogel adhesive reamain a significant challenge.

In contrast to the traditional method of using binary solvents to induce phase separation in homopolymeric hydrogels, we propose an alternative approach to fabricate underwater hydrogel adhesives by evoking phase separation in copolymeric hydrogels using a sole solvent—water. The key to this strategy is the copolymerization of two monomers that exhibit contrasting affinity to water. A hydrophilic monomer, acrylic acid (AAc) and a hydrophobic monomer, 2‐ethylhexyl acrylate (EHA), are taken as typical examples to validate the strategy. To fabricate a single‐phase copolymeric hydrogel, we employ dimethyl sulfoxide (DMSO) as a co‐solvent for the two monomers during copolymerization. Subsequently, phase separation is induced by equilibrating the copolymeric hydrogel in water, resulting in polymer‐dilute phases composed of PAAc and polymer‐rich phases composed of PEHA. This PS structure enhances underwater adhesion in two aspects. First, the hydrogel exhibits exceptional swelling resistance due to the enhanced elastic retractive force provided by the aggregation of hydrophobic PEHA chains, which inhibits network expansion. As a result, the hydrogel demonstrates a considerably low swelling ratio of ≈1 g g^−1^ and maintains stable mechanical properties during long‐term immersion in various aqueous environments, including water, seawater, and acid water. Second, the surface of the hydrogel possesses a large water contact angle (>90°), facilitating interfacial contact by enabling fast water drainage at the gel‐substrate interface. Consequently, the PS hydrogel exhibits strong, long‐lasting, reversible, and universal underwater adhesion to a wide range of soft and hard surfaces, creating opportunities for its application in underwater electronics. Furthermore, by introducing a small amount of carboxylic multiwalled carbon nanotubes (MWCNT‐COOH) into the PS hydrogel system, we demonstrate the potential application of this hydrogel adhesive as a key component in underwater sensing, communication, and triboelectric nanogenerators (TENG). We believe that this work sheds light on the development of next‐generation soft materials designed for aqueous conditions.

## 2 Results and Discussion

### 2.1 The Strategy and System

To validate efficacy of the water‐induced phase separation strategy, we employed acrylic acid (AAc) as a hydrophilic monomer and 2‐ethylhexyl acrylate (EHA) as a hydrophobic monomer to construct the initial network structure. A small amount of tannic acid (TA) was introduced into the system to facilitate underwater adhesion.^[^ ^20^ ^]^ The resulting P(AAc‐co‐EHA)/TA hydrogel, denoted as PAE_x_/T_y_, was named based on the feed ratio, where *x* represents the molar ratio of EHA to AAc and *y* indicates the mass of TA in the precursor solution. For all tests in this study, the representative phase‐separated (PS) sample used was the PAE_1/3_/T_20_ hydrogel, which was called PAE_1/3_/T for short. The non‐phase‐separated (NPS) hydrogel was also prepared for comparison by incorporating TA into a chemically crosslinked PAAc network (i.e., without hydrophobic polymers), which was named PAE_0_/T.

**Figure 1:**
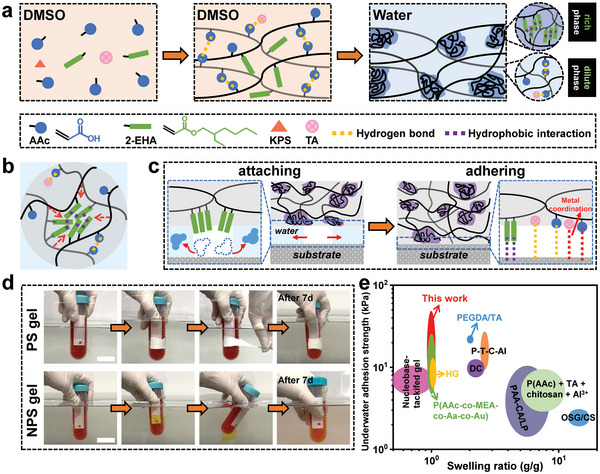
Water‐induced phase separation strategy for fabricating anti‐swelling hydrogel adhesives for underwater applications. a) Schematic illustration of the water‐induced phase separation method. b) Schematic depiction of the mechanism underlying the swelling resistance of the PS hydrogel. c) Mechanism of underwater adhesion of the PS hydrogel. d) Comparison of the underwater adhesion capability between the PS and NPS hydrogels. Scale bars represent 30 mm. e) Systematic comparison of underwater adhesion strength and swelling ratio between the state‐of‐the‐art hydrogels and the PS hydrogels developed in this study. Nucleobase‐tackifed gel: poly(acrylic acid‐co‐methoxyethyl acrylate‐co‐acrylated adenine); HG: poly (acrylic acid‐co‐methoxyethyl acrylate)/graphene; P(AAc‐co‐MEA‐co‐Aa‐co‐Au): poly(acrylic acid‐co‐methoxyethyl acrylate‐co‐acrylated adenine‐co‐acrylated uracil); DC: tetra‐armed polyethylene glycol; PEGDA/TA: polyethylene glycol diacrylate/tannic acid; P‐T‐C‐Al: poly(acrylic acid‐co‐N‐hydroxysuccinimide ester)‐tea polyphenol‐chitosan‐Al^3+^; PAA‐CA/LP: polyallylamine‐hydrocaffeic acid/laponite; P(AAc) + TA + chitosan + Al^3+^: polyacrylic acid + tannic acid + chitosan + Al^3+^; OSG/CS: oxidized succinoglycan/chitosan.

The water‐induced phase separation method, as illustrated in **Figure** **1**a, aimed to achieve two main features in the PS hydrogel system: swelling resistance and underwater adhesion. Initially, copolymerization of the two monomers, AAc and EHA, was conducted in a co‐solvent, dimethyl sulfoxide (DMSO), to generate an organogel with a homogenous network. Note that the preparation of the organogel involved the complete absence of chemical cross‐linkers, a characteristic indicative of its reliance on a fully physically cross‐linked network. Notably, the necessity of introducing a chemical crosslinker in the fabrication of the EHA‐free PAE_0_/T gel (as detailed in the Experimental Section) emphasized the pivotal role of EHA in facilitating the physical crosslinking process. We anticipated that there were two crucial functions of EHA in physically cross‐linking the organogel. On one hand, the presence of PEHA segments, characterized by long side chains, was prone to engender entanglement and the subsequent formation of physical crosslinking points during the thermal polymerization process. Second, the incorporation of electroneutral PEHA segments could counteract the mutual repulsion among PAAc chains, thereby promoting an enhanced formation of hydrogen bonds in cross‐linking the network. Subsequently, phase separation was induced by equilibrating the organogel in water for solvent exchange, taking advantage of the contrasting affinity of PAAc and PEHA to water. The hydrophobic PEHA segments in the gel network tended to aggregate into polymer‐rich phases due to their low affinity for the solvent, resulting in a considerably high polymer volume fraction that induces hydrophobic association.^[^ ^21^ ^]^ In contrast, the PAAc chains in water remained unfolded and formed dilute polymer phases, which possessed a relatively low modulus and polymer volume fraction. Under aqueous conditions, a competition arose between the polymer‐water interaction force and the elastic retractive force within the hydrogel (Figure 1b).^[^ ^22^ ^]^ The former was contributed by the carboxyl groups of PAAc, promoting swelling, while the latter stemmed from the hydrophobic association of the long side chains of PEHA, which resists swelling. Therefore, by precisely controlling the composition of hydrophilic and hydrophobic polymers in the hydrogel, the first objective of achieving anti‐swelling ability could be realized after phase separation. On the other side, the second goal, underwater adhesion, relied on the presence of rich hydrophobic polymer regions at the gel surface (Figure 1c). These regions facilitated water drainage at the gel‐substrate interface^[^ ^23^ ^]^ and generated numerous bonding sites for interfacial bridging, including hydrophobic interaction, hydrogen bonding, metal coordination, etc.^[^ ^11^, ^24^ ^]^ These features contributed to the enhanced underwater adhesion properties of the PS hydrogel.

A preliminary experiment was conducted to highlight the exceptional swelling resistance and underwater adhesion of the PS hydrogel. For comparison, the NPS hydrogel, dyed with bromocresol green, was utilized. As demonstrated in Figure 1d and Movie S1 (Supporting Information), a piece of PS hydrogel demonstrated the ability to repair a hole in a centrifuge tube through robust gel‐plastic adhesion, exhibiting remarkable resistance to underwater deformation. After 7 days, no obvious changes in size were observed in the PS gel and no liquid leaked from the perforated centrifuge tube. These findings substantiated the reliable anti‐swelling and adhesion capabilities of the PS hydrogel in underwater conditions. In contrast, the NPS hydrogel quickly detached from the centrifuge tube underwater upon slight movement, failing to prevent the liquid leakage. As a consequence, after 7 days, the water in the container became seriously contaminated by the liquid from the centrifuge tube. Quantitative measurements using the lap‐shear test showed that the underwater adhesion strength of the PS hydrogel was much greater than that of the NPS gel (Figure S1, Supporting Information). This simple demonstration underscored the significance of the water‐induced phase separation method in conferring favorable swelling resistance and underwater adhesion to hydrogels. That is, the formation of hydrophobic regions effectively drained water molecules between the gel‐substrate interface, creating a water‐poor environment that enhanced the interaction of adhesion groups with the substrate. Furthermore, a reasonable comparison between the PS hydrogels developed in this study and state‐of‐the‐art underwater hydrogel adhesives was conducted by plotting the underwater adhesion strength against the swelling ratio (Figure 1e, P(AAc)+TA+chitosan+Al^3+^: Ref. 20; PEGDA/TA: Ref. 25a; P‐T‐C‐Al: Ref. 25b; OSG/CS: Ref. 25c; PAA‐CA/LP: Ref. 25d; Nucleobase‐tackifed gel: Ref. 25e; HG: Ref. 25f; P(AAc‐co‐MEA‐co‐Aa‐co‐Au): Ref. 25g; DC: Ref. 25h).^[^ ^20^, ^25^ ^]^ Notably, the hydrogels manufactured through water‐induced phase separation occupied the upper‐left corner of the plot, indicating an excellent combination of high adhesion strength and low swelling ratio. The PS hydrogel also exhibited advantages over typical ionogels based on ionic liquids.^[^ ^26^ ^]^ On one hand, the PS hydrogel system demonstrated high compatibility with the aqueous environment in which it was applied, alleviating concerns about internal solvent leakage as often encountered with ionogels. On the other hand, the PS hydrogel offered ease of preparation and cost‐effectiveness compared to ionogels. Unlike ionogels, which often necessitated the synthesis of ionic liquids before the gel preparation, our approach required a straightforward one‐pot polymerization and solvent exchange to create the PS hydrogel. A comparative analysis of gel costs in Table S1 (Supporting Information) further accentuated the cost‐efficiency of the PS hydrogel in this study.

### 2.2 Structure Analysis

**Figure 2:**
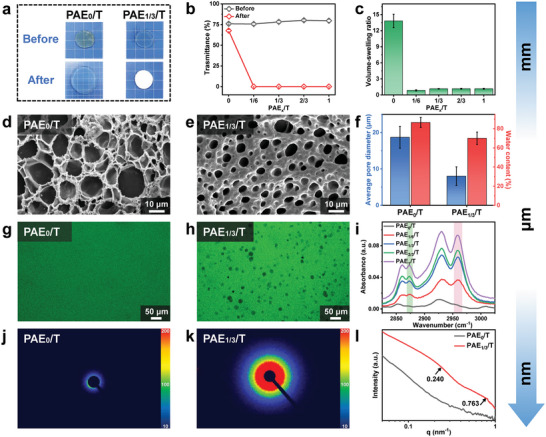
Analysis of the hydrogel structure from macroscopic to nanoscale. a) Photographs depicting the NPS PAE_0_/T gel and the PS PAE_1/3_/T gel before and after solvent exchange from DMSO to water. The background grid size is 5 mm. b) Transmittance of all PAE_x_/T gels before and after the solvent exchange. c) Volume‐swelling ratio of all PAE_x_/T gels. d) SEM image of the NPS PAE_0_/T gel. e) SEM image of the PS PAE_1/3_/T gel. f) Comparison of average pore diameter and water content between the PAE_0_/T gel and the PAE_1/3_/T gel. g) CLSM image of the NPS PAE_0_/T gel. h) CLSM image of the PS PAE_1/3_/T gel. i) Local ATR FT‐IR spectra of all PAE_x_/T gels. j) 2D SAXS patten of the NPS PAE_0_/T gel. k) 2D SAXS patten of the PS PAE_1/3_/T gel. l) 1D scattering intensity profiles of the PAE_0_/T gel and the PAE_1/3_/T gel, respectively.

The phase separation behavior of PEHA‐containing hydrogels was characterized and discussed in this section, spanning from a macroscopic view to a microcosm, with the NPS PAE_0_/T hydrogel serving as a comparison. The phase separation process was initiated by exchanging the solvent from DMSO to water, and the changes in size and appearance of the hydrogels before and after the immersion in water were recorded. As shown in **Figure** **2**a, the PEHA‐free organogel (PAE_0_/T) underwent significant swelling while maintaining high transparency during solvent exchange, attributed to the strong affinity of PAAc chains for water. In other words, when PEHA was not introduced into the gel system, there was no phase separation during the solvent exchange process, and immersion in water simply resulted in the dilution of the polymer chains. In contrast, the PEHA‐containing organogel (PAE_1/3_/T) became opaque upon immersion in water. Meanwhile, no significant size change was observed for the gel before and after solvent exchange. Attenuated total reflection Fourier‐transform infrared (ATR FT‐IR) and 1H NMR spectroscopies confirmed the absence of residual DMSO in the PAE_1/3_/T hydrogel after solvent exchange. As identified in Figure S2a (Supporting Information), the characteristic peak at 1019 cm^−1^, 947 cm^−1^ representing the S = O group in DMSO disappeared after solvent exchange for the PAE_1/3_/T gel,^[^ ^27^ ^]^ providing evidence of complete expulsion of DMSO from the network structure. The 1H NMR spectra further supported the ATR FT‐IR results by indicating the emergence of a solvent peak at 4.89 ppm and the disappearance of the DMSO peak at 2.71 ppm after solvent exchange (Figure S2b, Supporting Information). The changes in the transparency of hydrogels with different PEHA contents before and after solvent exchange were summarized in Figure 2b. For the PAE_0_/T gel, swelling caused a volume expansion of the hydrogel, resulting in a slight decrease in transmittance after water immersion. In contrast, all hydrogels containing PEHA exhibited a sharp decline in transmittance after solvent exchange due to the formation of a PS structure with a domain size larger than the wavelength of visible light. The volume‐swelling ratio of gels after solvent exchange further confirmed the critical role of PEHA in inducing phase separation for swelling resistance (Figure 2c). A point that was worth noting was that the volume swelling ratio exhibited a slight increases with the rise in hydrophobic monomer content. This observed phenomenon, while seemingly counterintuitive, could be attributed to the interplay between the surface and interior dynamics within the gel. When the hydrophobic monomer content was relatively low (x = 1/6, 1/3), the weaker hydrophobic interactions resulted in fewer hydrophobic groups migrating into the interior of the gel. Consequently, a larger proportion of hydrophobic groups remained at the gel surface, contributing to the formation of hydrophobic domains that in turn impede the diffusion of water molecules into the gel. Conversely, in the scenario where the hydrophobic monomer content was higher (x = 2/3, 1), the intensified hydrophobic interactions encouraged a greater migration of hydrophobic groups toward the interior of the gel. As a result, a relatively larger fraction of hydrophilic groups became exposed on the gel surface, which readily bound with water molecules, leading to a more pronounced swelling trend on the gel surface compared to the anti‐swelling effect experienced within the interior of the gel. From a macroscopic perspective, these results affirmed that the introduction of the hydrophobic polymer enables water‐induced phase separation behavior in the hydrogel.

On the microscopic scale, we investigated the impact of water‐induced phase separation on the microstructure of the hydrogel. Scanning electron microscopy (SEM) was employed to examine the gel morphology after solvent exchange. Figure 2d revealed that the PAE_0_/T hydrogel possessed a relatively loose network structure, characterized by large pore diameters. However, upon introducing hydrophobic segments into the system, the PAE_1/3_/T hydrogel exhibited a much more compact structure (Figure 2e). The comparison of average pore diameters and water content of the two hydrogels was presented in Figure 2f (The calculation method for the average pore diameter is shown in Figure S3, Supporting Information), which correlates well with the macroscopic size change of the hydrogels shown in Figure 2a. To provide further evidence, confocal laser scanning microscopy (CLSM) was utilized. In order to distinguish the polymer‐rich (PEHA) and polymer‐dilute (PAAc) phases in the PS hydrogel, a fluorescent dye (5‐carboxyfluorescein) specific to carboxyl groups was added. For the PAE_0_/T hydrogel, the CLSM image displays a uniform green color (Figure 2g), indicating the homogeneous distribution of carboxyl groups and a non‐phase‐separated structure. Interestingly, the CLSM image of the PAE_1/3_/T hydrogel exhibits two distinct regions: a bright region and a dark region (Figure 2h). Since PEHA does not contain any carboxyl groups and cannot be labeled by the fluorescent dye, it can be reasonably inferred that the dark areas with an average diameter of 20 µm represent the aggregation of hydrophobic polymer chains. These results provided direct evidence for the phase separation behavior of the PAE_1/3_/T hydrogel.

Further characterizations on the molecular scale were conducted to investigate the water‐induced phase separation behavior of the hydrogel. ATR FT‐IR spectra were employed to analyze the hydrogel compositions, as shown and discussed in Figure S4 (Supporting Information). Local ATR FT‐IR spectra focusing on the characteristic peaks of ‐CH_3_ groups of PEHA ≈2960 and 2870 cm^−1^ were obtained for various PAE_x_/T hydrogels (Figure 2i). It was observed that the ATR FT‐IR spectra of the PAE_0_/T hydrogel did not exhibit the characteristic peaks. Conversely, the maximum absorbance of these two groups increased with the elevation of the hydrophobic polymer content, indicating an increased hydrophobic polymer chain density in the gel after phase separation. This result provided evidence for the formation of polymer‐rich phases.^[^ ^28^ ^]^ Additionally, X‐ray Diffraction (XRD) patterns were analyzed to serve as auxiliary evidence for phase separation (Figure S5, Supporting Information). The XRD profile of the PAE_0_/T gel displayed a broad featureless peak at 30.3‐40.6°, attributed to the amorphous nature of PAAc. A similar peak was observed in the XRD profile of the PAE_1/3_/T gel, which shifted to 10.3‐27.3°. Applying Bragg's Law,^[^ ^29^ ^]^ the average spacing among carboxyl groups was calculated. The results indicated that the PAE_1/3_/T gel (4.01, 10.55 nm) exhibited a larger value compared to the PAE_0_/T gel (2.73, 3.62 nm), suggesting the aggregation of hydrophobic chains resulting in the separation of hydrophilic phases and an increased average distance among carboxyl groups. To investigate the structural difference between the NPS and PS hydrogels, Small‐Angle X‐ray Scattering (SAXS) measurements were performed. Figure 2j and Figure 2k showed the absence of a scattering ring for the NPS gel, while the PS gel exhibited a circular diffraction ring. This indicated that the NPS gel did not display a phase‐separated structure within the observed length range.^[^ ^30^ ^]^ Moreover, no correlation peaks were observed for the NPS gel, whereas wide scattering peaks (q = 0.240, 0.463 nm^−1^) were present for the PS gel in the 1D scattering intensity profiles (Figure 2l), suggesting a disparity in their network structures.^[^ ^31^ ^]^ Collectively, the aforementioned characterizations provide compelling evidence that the introduction of hydrophobic PEHA into the PAAc hydrogel system leads to water‐induced phase separation behavior.

### 2.3 Anti‐Swelling Ability

**Figure 3:**
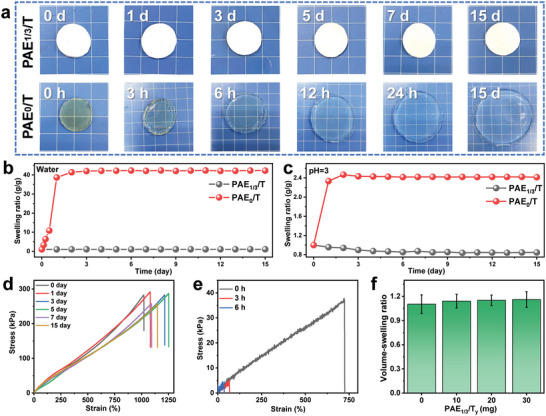
Exceptional anti‐swelling ability of the PS hydrogel. a) Photographs showing the size evolution of the PAE_1/3_/T and PAE_0_/T gels immersed in deionized water. The background grid size is 5 mm. b) Swelling ratio recorded for the PAE_1/3_/T and PAE_0_/T gels during a 15‐day immersion in deionized water. c) Swelling ratio recorded for the PAE_1/3_/T the PAE_0_/T gels during a 15‐day immersion in a solvent with pH=3. d) Tensile stress–strain curves of the PAE_1/3_/T gels after various immersion times in deionized water. e) Tensile stress–strain curves of the PAE_0_/T gel after various immersion times in deionized water. f) Volume‐swelling ratio of PAE_1/3_/T_y_ gels with different TA contents.

The phase separation phenomenon observed in the PEHA‐containing hydrogels contributed to the aggregation of hydrophobic chains, thereby enhancing their elastic retractive force and resistance to swelling. To substantiate this claim, a quantitative investigation of the swelling behavior of the hydrogels was conducted in this section. **Figure** **3**a presented a visual comparison of the swelling behavior between the PS PAE_1/3_/T and NPS PAE_0_/T hydrogels immersed in water. It was evident that the PAE_1/3_/T hydrogel showed no significant size change even after 15 days of immersion, while the PAE_0_/T hydrogel exhibited substantial swelling. Real‐time tracking of the swelling ratio further confirmed this observation, revealing that the weight of the PAE_1/3_/T hydrogel increased by only ≈3% at equilibrium (Figure 3b). In contrast, the PAE_0_/T hydrogel quickly reached a high swelling ratio of ≈40 g g^−1^. These distinct behaviors clearly demonstrated the crucial role of the PS structure in conferring anti‐swelling capability to the hydrogel. Notably, the PS hydrogel also exhibited resistance to swelling when exposed to other aqueous solutions such as acid solutions, artificial seawater, and organic solvents (Figure 3c; Figure S6, Supporting Information), with a low swelling ratio of ≈1 g g^−1^. The exceptional swelling resistance of the PS hydrogel primarily stemmed from the protonation of the carboxyl groups on the PAAc chains (in acid solutions), the hydrophobic association of side chains of PEHA segments (in artificial seawater), and the hydrophilic association of the PAAc chains (in organic solvents), respectively. Conversely, the NPS hydrogel consistently displayed weaker swelling resistance compared to the PS hydrogel due to the absence of hydrophobic aggregation that provided the elastic retractive force (Figure 3c; Figure S7, Supporting Information).

Further evidence of the swelling resistance of the PS hydrogel was provided through mechanical tests. Uniaxial tensile tests conducted on the PAE_1/3_/T hydrogel in water revealed its stable mechanical properties at different swelling times, consistent with the swelling results (Figure 3d). In comparison, the PAE_0_/T hydrogel exhibited inferior mechanical performance compared to the PAE_1/3_/T hydrogel and experienced a significant weakening of strength and elongation after swelling for several hours (Figure 3e). After swelling for over 6 h, the PAE_0_/T hydrogel became too fragile to be tested due to its significant water absorption. Notably, the PAE_1/3_/T hydrogel demonstrated slightly enhanced mechanical properties during swelling in seawater (Figure S8, Supporting Information), potentially attributed to the Hofmeister effect induced by salt ions (especially SO_4_^2−^ and Na^+^), which promoted the aggregation of PAAc chains.^[^ ^32^ ^]^ The influence of TA content on the swelling ratio was depicted in Figure 3f, showing that the addition of TA had minimal impact on the swelling behavior of the PS PAE_1/3_/T hydrogel. These results confirmed that phase separation induced exceptional swelling resistance of the hydrogel under various aqueous conditions.

### 2.4 Underwater Adhesion Performance

**Figure 4:**
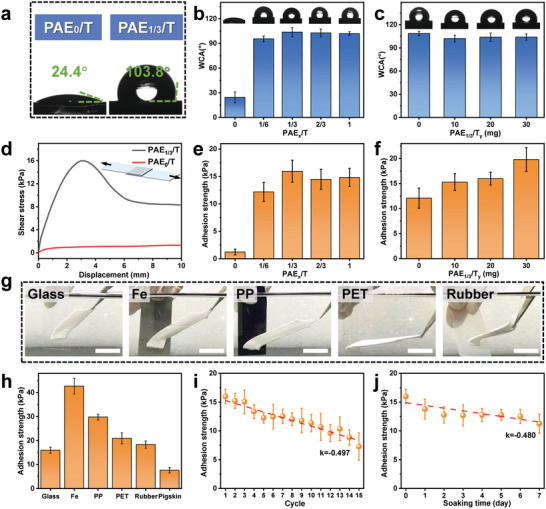
Underwater adhesion performance of the PS hydrogel. a) Comparison of the WCA between the PAE_0_/T and the PAE_1/3_/T hydrogels. b) Influence of PEHA content on the WCA of PAE_x_/T hydrogels. c) Influence of the TA content on the WCA of PAE_1/3_/T_y_ hydrogels. d) Shear stress‐displacement curves obtained from lap‐shear tests, measuring the adhesion performance of the PAE_0_/T and PAE_1/3_/T hydrogels on a glass plate substrate. e) Effect of PEHA content on the shear adhesion strength of PAEx/T hydrogels to glass. f) Effect of TA content on the shear adhesion strength of PAE_1/3_/T_y_ hydrogels to glass. g) Photographic evidence demonstrating the universal underwater adhesion of the PAE_1/3_/T gel to different substrates. Scale bars represent 25 mm. h) Summary of the shear adhesion strength of the PAE_1/3_/T gel to different substrates underwater. i) Evolution of the shear adhesion strength of the PAE_1/3_/T gel underwater with repeated cycles. j) Shear adhesion strength of the PAE_1/3_/T gel after immersion in water for varying time periods.

The phase‐separated structure results in strong resistance of the PAE_1/3_/T hydrogel to swelling, allowing it to maintain structure stability under aqueous conditions and promising its application as underwater adhesives.^[^ ^33^ ^]^ Common hydrogels face challenges in underwater adhesion due to the formation of a water layer at the gel‐substrate interface caused by the strong hydration ability of hydrophilic polymer strands, hindering the formation of interfacial molecular bridges.^[^ ^34^ ^]^ By incorporating hydrophobic polymers into the hydrogel system, the affinity of the gel surface to water was weakened, facilitating water drainage at the gel‐substrate interface.^[^ ^35^ ^]^ In other words, water‐induced phase separation promoted underwater adhesion by creating surface polymer domains enriched with hydrophobic chains, as opposed to the predominantly dilute hydrophilic chains on the non‐phase‐separated hydrogel surface. A representative comparison of the water contact angle (WCA) between the PAE_1/3_/T and PAE_0_/T hydrogels was shown in **Figure** **4**a. The PS hydrogel, with hydrophobic domains resulting from phase separation, exhibited a significantly larger WCA of 103.8° compared to the NPS hydrogel (24.4°). This result indicated that the aggregation of PEHA chains at the gel surface altered its surficial nature from hydrophilic to hydrophobic, facilitating water drainage and favoring underwater adhesion. Figure 4b illustrated the influence of PEHA content on the surface properties of the PAE/T hydrogels. All PEHA‐containing hydrogels demonstrated similar WCAs, significantly higher than that of the PEHA‐free hydrogel, highlighting the crucial role of phase separation in creating a relatively hydrophobic surface. Interestingly, the WCA of the PS hydrogel did not exhibit a linear increase with the increment of the PEHA content. Instead, it initially increased and then decreased. This phenomenon could be understood as follows. Initially, with the increase in hydrophobic monomer content, the WCA of the hydrogel surface increased. This could be attributed to the introduction of hydrophobic moieties, which tended to migrate towards the air interface, enhancing the surface hydrophobicity. However, as the hydrophobic monomer content continued to increase, a point was reached where the internal hydrophobic interactions within the gel matrix became more significant. This led to a greater migration of hydrophobic molecular chains into the interior of the gel, rather than remaining on the surface. Consequently, the surface became enriched with hydrophilic segments, leading to a decrease in the WCA. It was important to note that TA, a hydrophilic component, was also present in the hydrogel system, which could potentially compromise the hydrophobicity of the gel surface. However, as depicted in Figure 4c, the addition of TA resulted in only a slight reduction in WCA, which still remained above 90°. Therefore, it was primarily the phase separation through the introduction of PEHA that predominantly influenced the surface properties of the hydrogel system.

To further confirm the advantageous role of phase separation in enhancing underwater adhesion, lap‐shear tests were conducted using a glass plate as the substrate to quantify the adhesion strength of the hydrogels. Figure 4d presented the shear stress‐displacement curves for the NPS PAE_0_/T and PS PAE_1/3_/T hydrogels. The PS hydrogel exhibited high adhesion strength underwater, reaching a peak value of 16 kPa. In contrast, the NPS hydrogel showed almost no adhesion in water. These distinct adhesion behaviors could be attributed to the contrasting surface properties of the two hydrogels. The hydrophobic polymer domains within the PS hydrogel facilitated water drainage at the gel‐substrate interface and provided numerous bonding sites for adhesive groups. Conversely, the interfacial water layer inhibited potential interactions between adhesive groups of the NPS hydrogel and the substrate, leading to limited adhesion.^[^ ^18^ ^]^

The aforementioned results indicated that the phase‐separated structure promoted interfacial interaction between hydrogels and substrates through efficient water drainage. However, achieving strong hydrogel adhesion typically requires a combination of robust interfacial interaction and high energy dissipation near the interface.^[^ ^36^ ^]^ Therefore, we conducted mechanical property evaluations on the PAE_1/3_/T and PAE_0_/T hydrogels. Uniaxial tensile tests were performed, revealing significant advantages of the PS hydrogel over the NPS gel in terms of fracture stress, fracture strain, work of extension, and Young's modulus (Figure S9a, Supporting Information). Furthermore, we assessed the energy dissipation capability by subjecting the hydrogels to cyclic loading and unloading at a fixed strain of 400% (Figure S9b, Supporting Information). The PS hydrogel exhibited pronounced hysteresis during the loading‐unloading cycle, indicating its favorable energy dissipation capability. That is, when a crack at the PS gel‐substrate interface tends to propagate, polymer chains at the crack front undergo stretching, transferring the load from the crack front to the bulk and fracturing sacrificial bonds over a large zone. Consequently, the total dissipated energy comprises the bond energy of a densely packed polymer chain layer at the gel‐substrate surface and the hysteresis of the PS gel near the interface. This synergistic effect contributes to the tough adhesion of the PS hydrogel underwater. In contrast, the nearly overlapping loading‐unloading stress–strain curves of the NPS hydrogel signified its non‐dissipative nature. Additionally, rheological tests confirmed that the incorporation of PEHA transformed the original NPS hydrogel from an elastic network to a viscoelastic one, which promoted energy dissipation (Figure S10, Supporting Information).

To optimize the underwater adhesion of the PS hydrogel, we investigated the influence of gel composition on its adhesion performance. Figure 4e and Figure S11 (Supporting Information) demonstrated the effect of hydrophobic polymer content on the underwater adhesion strength, which initially increased significantly and then slightly decreased with increasing PEHA content. The substantial increase in adhesion strength confirmed the crucial role of the PS structure in conferring underwater adhesion capability to the hydrogel. The slight decline in adhesion strength could be explained as follows. Since all PEHA‐containing hydrogels possessed a hydrophobic surface, the fluctuation in adhesion strength can be attributed to the trade‐off between energy dissipation and effective contact area of the gel. The former was reflected by the mechanical hysteresis during a loading‐unloading cycle, while the latter was closely related to Young's modulus of the hydrogel.^[^ ^37^ ^]^ Before reaching 2/3 (mol mol^−1^) of the PAAc, the energy dissipation capability was significantly enhanced (Figure S12a,b, Supporting Information). At the same time, the Young's modulus of the hydrogel with such PEHA contents remained relatively low, ensuring effective contact with the substrate for interfacial bridging (Figure S12c,d, Supporting Information). Therefore, the improved energy dissipation ability primarily contributed to enhanced adhesion performance at low PEHA contents. However, when the PEHA content exceeded 2/3 (mol mol^−1^) of the PAAc, the Young's modulus underwent a sharp increase, which could reduce the effective contact area and the number of bonding sites for the hydrogel to transfer stress from the crack front to the bulk. Consequently, the adhesion strength was compromised. Adjusting the content of hydrophilic monomer also impacted the underwater adhesion strength of the hydrogel. As shown in Figure S13a,b (Supporting Information), elevating the AAc concentration to 3.2 M enhanced the adhesion strength of the gel to pigskin, reaching ≈11 kPa. However, an excessive inclusion of hydrophilic monomer could disrupt the hydrophilic‐hydrophobic equilibrium, causing gel swelling and consequent reduction in underwater adhesion.

In addition to the influence of monomer content, we also investigated the impact of TA content on the underwater adhesion ability of the hydrogels. Interestingly, unlike the trend observed with changing PEHA content, the adhesion strength of the PAE_1/3_/T_y_ hydrogel showed a monotonic improvement with increasing TA content (Figure 4f; Figure S14, Supporting Information). Examination of the mechanical properties of the PAE/T hydrogels with varying TA contents revealed a slight enhancement in hysteresis without significant changes in the Young's modulus (Figure S15, Supporting Information). Rheological analysis further indicated that the TA content had minimal effect on the viscoelasticity of the hydrogel, with only slight modifications in the storage and loss modulus (Figure S16, Supporting Information). In this scenario, the adhesion strength primarily depended on the energy dissipation capability of the hydrogel, as the interfacial condition (i.e., effective contact area) was assumed to be similar across the samples.

Furthermore, we evaluated the adhesion strength of all hydrogels in air for reference purposes. The summarized results can be found in Figure S17 (Supporting Information). Interestingly, the NPS PAE_0_/T hydrogel demonstrated relatively high adhesion strength in air, despite its non‐adhesive behavior underwater. This observation indirectly highlighted the importance of the phase‐separated structure in facilitating water drainage at the interface, which was crucial for achieving strong underwater adhesion.

The PS PAE_1/3_/T hydrogel exhibited robust and universal underwater adhesion to various substrates, regardless of their hydrophilicity or hydrophobicity, as well as their hardness. Figure 4g illustrated the firm adhesion of the PAE_1/3_/T hydrogel to glass, metal, plastics, rubber, and soft tissue underwater. Note that no pre‐gluing in air was required for the underwater adhesion of the hydrogel. It can directly lift the substrate (for example, the polyethylene terephthalate plate in Movie S2, Supporting Information) from the water by simple attachment. The interface of the gel‐substrate was imaged by an ultra‐depth of field microscope (Figure S18, Supporting Information), and no visible voids on the micron scale, indicating the excellent bonding of the gel to substrates. The hydrogel maintained strong adhesion even under significant deformation. The corresponding shear stress‐displacement curves and adhesion strength were shown in Figure 4h and Figure S19 (Supporting Information), respectively. The adhesion strength of the PAE_1/3_/T hydrogel reached over 40 kPa when in contact with an iron plate, and remained ≈6 kPa when adhering to pigskin, demonstrating its impressive underwater adhesion capability to diverse substrates. Similar trends were observed for the adhesion of the PAE_1/3_/T hydrogel to different substrates in air, as depicted in Figure S20 (Supporting Information). The relatively high adhesion strength of the hydrogel to the iron plate, in comparison to that of other substrates, could be attributed to the formation of metal coordination bonds between this substrate and the carboxylic acid groups from PAAc, as well as the catechol groups from TA. Such a physical interaction boasted higher bond energy in contrast to hydrogen bonding or hydrophobic interactions,^[^ ^38^ ^]^ which primarily facilitated the adhesion between the gel and other substrates such as rubber, plastic, and pigskin. To provide a lucid comprehension of the adhesion mechanism, the molecular interactions between the hydrogel and diverse substrates are illustrated in Figure S21 (Supporting Information).

We also investigated the durability of the underwater adhesion. Repeatability tests revealed slightly deteriorated shear stress‐displacement curves and adhesion strength for the PAE_1/3_/T hydrogel (Figure 4i; Figure S22a, Supporting Information), possibly due to the reduction of effective contact area caused by the wrinkling of the gel surface (Figure S22b, Supporting Information). The oxidation of phenol hydroxyl groups in TA could be another contributing factor, as evidenced by the noticeable decrease in adhesion strength of the PAE_1/3_/T hydrogel in air after 15 cycles (Figure S23, Supporting Information). To evaluate the stability of underwater adhesion, we examined the effect of soaking time on the adhesion strength. Due to the excellent resistance of the PS PAE_1/3_/T hydrogel to swelling in water, the soaking time had minimal impact on its adhesion strength. Even after 7 days of immersion, the hydrogel maintained comparable adhesion strength to its initial state (Figure 4j; Figure S24, Supporting Information). In contrast, the underwater adhesion strength of the NPS PAE_0_/T gel to glass, as observed across different repeated cycles (Figure S25, Supporting Information) and durations of soaking (Figure S26, Supporting Information), consistently remained below 2 kPa. This effectively indicates that the PAE_0_/T gel, due to the lack of hydrophobic domains on its surface, exhibits almost no adhesion in water. Meanwhile, since the PAE_0_/T gel showed a rather high swelling ratio in water, the measurement of underwater adhesion strength was limited to conditions involving 4 cycles and short soaking durations. The contrasting results between the PS PAE_1/3_/T gel and the NPS PAE_0_/T gel underscored again the critical significance of hydrophobic domains in conferring anti‐swelling and underwater adhesion attributes.

Next, we attempt to investigate the external factors influencing the underwater adhesion of the PS gel, including parameters such as the duration of solvent exchange, water temperature, preload, solvent type, and hydrophobic monomer type. As demonstrated in Figure S27a,b (Supporting Information), the adhesion strength exhibited an initial increase followed by stabilization with prolonged soaking time. This phenomenon could be attributed to the progressive phase separation process, leading to the gradual formation of hydrophobic domains on the gel surface. Consequently, the hydrophobicity of the PS gel surface intensified as the duration of solvent exchange extended, contributing to a remarkable enhancement in underwater adhesion strength. Notably, the phase separation process tended to be completed within ≈180 min, as evidenced by stabilization in WCA over different time intervals (Figure S27c, Supporting Information).

Water temperature also significantly influenced the underwater behavior of the PS hydrogel. With increasing water temperature, the underwater adhesion strength first experienced an increment followed by stabilization (Figure S28, Supporting Information). In low‐water water, the freezing of the PS gel resulted in a substantial enhancement of the stiffness, causing a reduction in the effective contact area and the number of bonding sites available for stress transfer from the crack front to the bulk.^[^ ^39^ ^]^ As the water temperature rose, the underwater adhesion strength underwent a slight increase. This could be attributed to the elevated temperature promoting molecular chain movement, thereby facilitating more interactions between the hydrogel and the substrate. As a result, for optimal performance, the PS gel should be utilized at temperatures exceeding 0 °C.

Preload was another influencing factor of the underwater adhesion for the PS hydrogel. To comprehensively explore its impact, we conducted experiments by varying the preload applied during the adhesion test. Specifically, we replaced the standard 100 g weight with different weights (0 g, 25 g, 50 g, 75 g, 120 g) and subsequently measured the resulting underwater adhesion strength (as depicted in Figure S29, Supporting Information). The findings revealed an interesting trend: as the preload increased, the underwater adhesion strength initially exhibited an upward trajectory, followed by reaching a state of stability. This behavior could be attributed to a specific mechanism. With an augmented preload, larger forces were exerted on the hydrogel, leading to the exclusion of water droplets from the gel‐substrate interface. Consequently, this process enhanced the effective contact area between the hydrogel and the substrate, thereby boosting underwater adhesion. However, this effect reached a saturation point once the effective contact area was maximized. Further elevating the preload beyond this saturation point did not lead to significant additional enhancement in adhesion strength.

Meanwhile, the combined hydrophobic and hydrophilic network system uniquely conferred the hydrogels with robust adhesion capability across various solvents, including water, seawater, acid solutions, toluene, hexane, and dodecane (Figure S30, Supporting Information). Notably, the adhesion strength was observed to be slightly lower in seawater and acidic solutions compared to water. In seawater, the presence of sodium cations from sodium chloride neutralized the negatively charged carboxylic acid groups, thereby weakening their adhesion potential. On the other hand, the ionization of hydrochloric acid in acidic solutions enhanced the protonation of carboxyl groups, causing certain PAAc molecular chains to aggregate. Consequently, this led to a reduction in the distribution of carboxyl groups on the gel surface. Furthermore, the hydrophilic domains within the PS gels effectively resisted the influence of organic solvents, substantiating their suitability for constructing adhesion in oil environments.

The type of hydrophobic monomer employed also exerted a significant influence on the underwater behavior of the hydrogel. This underscored the universal utility of the water‐induced phase separation methodology in crafting hydrogels with both anti‐swelling and underwater adhesion properties. To substantiate this, we conducted a comparative study by selecting three additional hydrophobic monomers: 2‐methoxyethyl acrylate (MEA), 2,2,2‐trifluoroethyl methacrylate (TFEA), and 2,2,3,4,4,4‐hexafluorobutyl 2‐methylprop‐2‐enoate (STAB). The structures of these hydrophobic monomers were shown in Figure S31a (Supporting Information). Subsequently, we prepared the corresponding PAM_1/3_/T, PAF_1/3/_T, and PAS_1/3_/T gels using the identical preparation protocol with that of the PAE_1/3_/T. Remarkably, the WCAs of the PAM_1/3_/T, PAF_1/3_/T, and PAS_1/3_/T gels were all below 90° (Figure S31b, Supporting Information), still distinctly surpassing the WCA of the NPS PAE_0_/T gel (24.4°). This result indicated the successful formation of hydrophobic domains across all gel surfaces. Compared with EHA, the hydrophobicity of MEA is relatively low, leading to the observed reduction in WCA of the PAM_1/3_/T gel. On the other hand, the abundant C‐F bonds in PAF_1/3_/T and PAS_1/3_/T gels tend to interact via hydrogen bonding with carboxyl groups, contributing to the decreased WCA. A comprehensive assessment of underwater adhesion strength across all gels firmly places the PAE_1/3_/T gel as the champion (Figure S31b,c, Supporting Information), affirming the pivotal role of a harmonious hydrophilic‐hydrophobic equilibrium in conferring potent underwater adhesion capabilities to the gel.

Given the outstanding adhesive capabilities of the PS PAE_1/3_/T hydrogel, it demonstrated remarkable potential as an instant sealing tape. To draw a comparison, a piece of NPS PAE_0_/T hydrogel was used. As depicted in Figure S32 and Movie S3 (Supporting Information), the PAE_1/3_/T gel effectively halted leakage from a perforated centrifuge tube, while the PAE_0_/T gel failed to adhere due to weak adhesion caused by the presence of a water layer at the interface. To assess the endurance of the hydrogel sealing tape in aquatic conditions, perforated tubes containing dye solutions were placed in a beaker filled with water. Figure S32a (Supporting Information) clearly showed that the water in the beaker remained uncontaminated even after the PS hydrogel‐sealed tube was immersed for 7 days. Corresponding UV–vis spectra provided additional verification, confirming the absence of any leakage from the contaminated solution into the beaker (Figure S32b, Supporting Information). In contrast, the PAE_0_/T gel quickly detached from the tube surface in water, resulting in significant pollution of the water inside the beaker within just 10 min (Figure S32c,d, Supporting Information).

### 2.5 Sensing Capability Underwater

**Figure 5:**
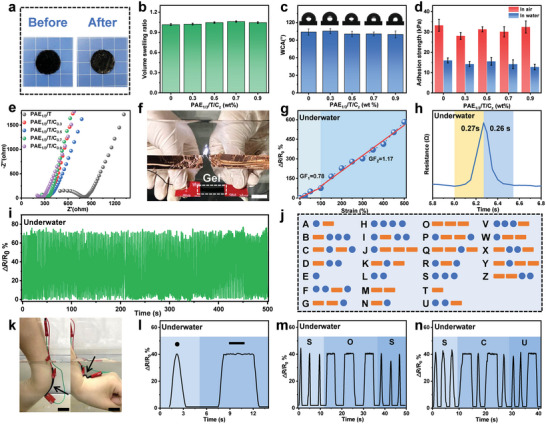
Sensing capability of the MWCNT‐COOH‐containing PS hydrogel underwater. a) Photographs depicting the PAE_1/3_/T/C_0.5_ gel before and after solvent exchange from DMSO to water. The background grid size is 5 mm. b) Effect of MWCNT‐COOH content on the volume‐swelling ratio of PAE_1/3_/T/C_z_ gels. c) Effect of MWCNT‐COOH content on the WCA of PAE_1/3_/T/C_z_ gels. d) Influence of MWCNT‐COOH content on the adhesion strength of PAE_1/3_/T/C_z_ gels to glass in both air and underwater conditions. e) EIS curves of PAE_1/3_/T/C_z_ gels with varying MWCNT‐COOH content. f) Photographs demonstrating the ability of the PAE_1/3_/T/C_0.5_ gel to conduct electricity and light up an LED bulb underwater, suggesting good underwater conductivity. The scale bar represents 30 mm. g) Relative resistance change of the PAE_1/3_/T/C_0.5_ gel as a function of strain underwater. h) Response and recovery time of the PAE_1/3_/T/C_0.5_ gel sensor underwater. i) Repetitive relative resistance change of the gel upon continuous loading and unloading cycles at a fixed strain of 100% underwater. j) Definition of Morse codes for information delivery. k) Photographs showcasing the deformability of the hydrogel sensor, demonstrating its ability to detect body motion. The scale bars represent 30 mm. l) Definition of “dot” and “dash” signals by controlling the deformation time of the gel. m,n) Information delivery using Morse. codes underwater.

The preceding sections had established the remarkable anti‐swelling capability and underwater adhesion performance of the PS hydrogel. In this section, we investigated the potential of PS hydrogel for underwater electronics by examining its sensing capability. To enhance the electrical conductivity of the gel, carboxylic multiwalled carbon nanotube (MWCNT‐COOH) was incorporated into the network. It was confirmed that this addition had minimal impact on the phase‐separation behavior and surface properties of the PS hydrogel. **Figure** **5**a illustrated that the diameter of the PAE_1/3_/T/C_0.5_ gel (see the experimental section for nomenclature) remained almost unchanged after the solvent exchange from DMSO to water, similar to the behavior observed in the PAE_1/3_/T gel shown in Figure 2a. Figure 5b demonstrated that the volume swelling ratio of the PS hydrogel remained relatively constant as the MWCNT‐COOH content increased, indicating that MWCNT‐COOH did not hinder the anti‐swelling ability of the gel. The ATR FT‐IR spectra of the PAE_1/3_/T and PAE_1/3_/T/C_0.5_ gels in Figure S33a (Supporting Information) both exhibited characteristic peaks corresponding to ‐OH and C = O groups. Furthermore, the CLSM image of the PAE_1/3_/T/C_0.5_ gel (Figure S33b, Supporting Information) displayed regions of light and dark, indicating a similar phase‐separated structure to that of the PAE_1/3_/T gel. Interestingly, the pore diameter of the PAE_1/3_/T/C_0.5_ gel was relatively small (≈5.2 µm, Figure S33c, Supporting Information) compared to the PAE_1/3_/T gel (Figure 2f). This could be attributed to the hydrogen bonding between MWCNT‐COOH and ‐COOH in the gel network, which caused the compaction of the structure. With regard to the surface properties, the WCA of the PS hydrogel slightly decreased with increasing MWCNT‐COOH content but remained above 90° (Figure 5c), indicating the preservation of hydrophobicity on the gel surface. Meanwhile, the introduction of MWCNT‐COOH had no significant effect on the adhesion strength of the hydrogel when a glass plate was used as the substrate, whether in air or underwater (Figure 5d; Figure S34, Supporting Information). The PAE_1/3_/T/C_0.5_ gel also exhibited universal adhesion capability, firmly adhering to various substrates (Figure S35, Supporting Information). Importantly, the gel readily adhered to human skin and demonstrated excellent compliance with body deformation in both air and underwater conditions. No residual hydrogel was observed on the body after removal, indicating strong and reversible adhesion. To gain further insight into the interface between the PAE_1/3_/T/C_0.5_ gel and soft tissues, we employed an ultra‐depth‐of‐field microscope and utilized pigskin as the substrate. On the micron scale, no visible voids at the gel‐skin interface were observed in both air and underwater conditions (Figure S36, Supporting Information), confirming the excellent bonding of the gel to substrates.

Peeling tests provided additional evidence for the good underwater adhesion capabilities of the hydrogel. By utilizing rubber and pigskin as examples, we performed 180^o^ peeling test to quantify the adhesion energy of the PAE_1/3_T/C_0.5_ hydrogel to these soft substrates. The results, as presented in Figures S37 and S38 (Supporting Information), demonstrated adhesion energy of 154 and 83.2 N m^−1^ for rubber and pigskin adhesion, respectively. Importantly, these values were comparable with those observed in elastomers adhering to various solids and commercial tissue adhesives,^[^ ^40^ ^]^ verifying the applicability of the PAE_1/3_T/C_0.5_ gel in underwater electronics.

We also explored the influence of MWCNT‐COOH on the mechanical properties of the PS hydrogel. Figure S39a (Supporting Information) showed that the fracture stress of the PAE_1/3_/T/C_z_ gel initially increased and then decreased with the increase of MWCNT‐COOH content. This observation suggested that the mechanical reinforcement was attributed to the hydrogen bonding between MWCNT‐COOH and carboxyl groups. However, excessive MWCNT‐COOH tended to aggregate and acted as defects in the network, leading to a weakening of the mechanical properties. Among all the MWCNT‐COOH‐containing PS hydrogels, the PAE_1/3_/T/C_0.5_ gel exhibited the optimal mechanical properties and was chosen as the representative sample for further tests. Rheological tests confirmed that all hydrogels exhibited viscoelastic behavior (Figure S39b, Supporting Information), indicating their desirable energy dissipation capability, which was beneficial for the adhesion performance. The loading‐unloading tensile test at different strains on the PAE_1/3_/T/C_0.5_ gel demonstrated that the hysteresis gradually increased with the applied strain (Figure S39c, Supporting Information). Additionally, the cyclic tensile test revealed that the hydrogel retained a certain amount of hysteresis even after undergoing continuous loading‐unloading cycles (Figure S39d, Supporting Information). These tests provided clear evidence of the satisfactory energy dissipation capability when MWCNT‐COOH is incorporated into the PS hydrogel. Due to the presence of numerous physical bonds in the network structure, the PS hydrogel containing MWCNT‐COOH also exhibited high self‐recoverability. The hysteresis of the hydrogel could fully recover with a resting time of 20 min (Figure S40a, Supporting Information). Moreover, two pieces of cut PS hydrogel could merge into a single intact piece at room temperature, demonstrating its self‐healing capability. Figure S40b (Supporting Information) showed that the mechanical properties of the gels gradually improved with longer healing times. The healing efficiency, defined as the work of extension ratio of the healed gel to the original one, reached ≈78% after 24 h of healing at room temperature (Figure S40c, Supporting Information). SEM analysis vividly illustrated that cracks in the hydrogel gradually disappeared after 24 h (Figure S41, Supporting Information). All of the above results validated that the introduction of MWCNT‐COOH did not compromise the anti‐swelling capability, underwater adhesion, and mechanical properties of the PS hydrogel. These findings opened up opportunities for utilizing the hydrogel in various underwater applications.

Next, we examined the influence of MWCNT‐COOH on the electrical conductivity of the PS hydrogel. MWCNT‐COOH is widely known for its excellent conductivity, so its incorporation should enhance the conductivity of the gel. Based on the Nyquist plot in Figure 5e, the electrical conductivity of the PAE_1/3_/T/C_0.5_ hydrogel was calculated to be 0.35 mS cm^−1^. The photograph in Figure 5f demonstrated that the PAE_1/3_/T/C_0.5_ hydrogel efficiently conducted electricity, as it successfully illuminated an LED bulb underwater. This observation confirmed its satisfactory conductivity under aqueous conditions. The self‐healing ability of the hydrogel enabled the immediate restoration of conductivity when two pieces of cut hydrogels were brought into contact, as shown in Figure S42a (Supporting Information). After a self‐healing time of just 10 min, the conductivity of the gel reached ≈99% of the initial value (Figure S42b,c, Supporting Information). Furthermore, increasing the MWCNT‐COOH content had a positive effect on the conductivity (Figure S43, Supporting Information), allowing for convenient adjustment of the overall properties of the hydrogel. Moreover, stability tests demonstrated that the PAE_1/3_/T/C_0.5_ hydrogel maintained nearly constant conductivity underwater for 7 days (Figure S44a, Supporting Information), thanks to its exceptional swelling resistance. When the hydrogel was immersed in artificial seawater, the conductivity was further enhanced (Figure S44b,c, Supporting Information), likely due to the influx of abundant free ions into the gel network. These results highlighted the PAE_1/3_/T/C_0.5_ hydrogel as an ideal candidate for underwater sensing applications.

Continuing with our study, we measured the gauge factor (GF) of the hydrogel underwater, which is an important parameter for evaluating its sensing capability. The GF is defined as the relative resistance variation per strain. The data fitting results were presented in Figure 5g. The hydrogel demonstrated a relatively high GF of 0.78 and 1.17 in the strain range of 0%–100% and 100%–500%, respectively. Additionally, the hydrogel exhibited a rapid response to external forces, with the ability to detect and respond to deformations within 0.27 s (Figure 5h). Given the high deformability and recoverability of the hydrogel, we also evaluated its repetitive sensing capability by monitoring its real‐time relative resistance variation during continuous loading‐unloading cycles at a fixed strain of 100%. The result in Figure 5i demonstrated that the gel sensor consistently produced stable output signals for each cycle, indicating its reliable and repeatable sensing performance.

Based on the remarkable underwater sensing capability of the PS hydrogel, we were excited to explore its potential application in conveying information through Morse codes. Morse code is a widely recognized character‐encoding scheme that utilizes electrical pulses represented as dots and dashes to send messages (Figure 5j).^[^ ^41^ ^]^ Leveraging the strong adhesion of the hydrogel to human skin and its high compliance with body motion (Figure 5k), we could define the transient resistance variation of the gel as the “dot” signal and the prolonged resistance variation as the “dash” signal by controlling the deformation time (Figure 5l). To demonstrate this concept, we conducted a proof‐of‐concept experiment using the PAE_1/3_/T/C_0.5_ hydrogel as the sensor to transmit short messages underwater, such as “SOS” and “SCU” (Figure 5m,n), as well as “UP,” “DOWN,” and “HELP” (Figure S45a–c, Supporting Information). By fixing the hydrogel on different body parts, such as the finger, wrist, or elbow, we were able to detect body movements underwater and obtain steady electrical signals during successive cycles (Figure S45d–f, Supporting Information).

### 2.6 Application to Underwater Triboelectric Nanogenerator

**Figure 6:**
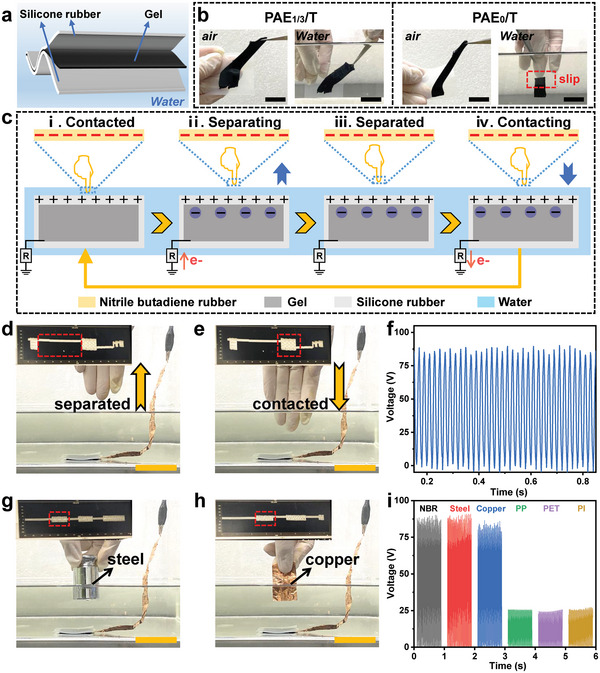
Application to underwater triboelectric nanogenerator. a) Schematic illustration of the TENG. b) Photographs showing the adhesion of the PAE_1/3_/T/C_0.5_ gel and the PAE_0_/T/C_0.5_ gel to silicone rubber in air and underwater. c) Schematic representation of the working principle of the underwater TENG. d) Photograph demonstrating the rubber glove separated the water surface, resulting in only noise signal output. e) Photograph displaying the rubber glove in contact with the water surface, generating a triboelectric signal output. f) Open‐circuit voltage of the TENG obtained by touching the water surface with the rubber glove. g) Photograph showing the steel in contact with the water surface, producing a triboelectric signal output. h) Photograph revealing copper in contact with the water surface, generating a triboelectric signal output. i) Open‐circuit voltage of the TENG obtained by touching the water surface with different materials. Scale bars in (b) represent 15 mm. Scale bars in (d), (e), (g), (h) represent 30 mm.

Due to the remarkable combination of anti‐swelling, adhesion, and sensing capabilities exhibited by the PS PAE_1/3_/T/C_0.5_ hydrogel, we proceeded to investigate its application in underwater triboelectric nanogenerator (TENG) systems. In this setup, two pieces of silicone rubber were employed as triboelectric materials, encapsulating the hydrogel, while the PAE_1/3_/T/C_0.5_ hydrogel served as the electrode to facilitate electron transport for the triboelectric charges generated in the silicone rubber (**Figure** **6**a).^[^ ^42^ ^]^ The photographs in Figure 6b vividly demonstrated the strong adhesion of the PAE_1/3_/T/C_0.5_ hydrogel to the silicone rubber, ensuring efficient electron transport between the two components. Conversely, the PAE_0_/T/C_0.5_ hydrogel exhibited good adhesion to the silicone rubber in air but failed to maintain its adhesion properties in water due to the weak drainage capability of interfacial water. Consequently, only the PS hydrogel could be utilized for constructing an effective underwater TENG system.

The working principle of the underwater TENG is depicted in Figure 6c. Initially, in state i, when a triboelectric material, such as a rubber glove, comes into contact with the water where the TENG is submerged, the triboelectric effect is induced, resulting in the generation of equal amounts of opposite charges on the surfaces of the rubber glove and silicone rubber. As the rubber glove begins to separate, the TENG enters state ii. Due to the incomplete shielding of the positive charge on the surface of the silicone rubber by the negative charge on the rubber glove surface, a neutralization process occurs, leading to the generation of an electron flow towards the hydrogel. In state iii, the rubber glove is completely separated from the water, and the movement of external electrons towards the hydrogel ceases as neutralization is achieved. Finally, in state iv, when the rubber glove approaches the silicone rubber, the potential difference between the two triboelectric materials decreases due to enhanced electrostatic shielding. This repels negative electrons on the surface of the hydrogel and facilitates the flow of excess electrons in the outer circuit to the ground. This working mode can be restarted from state i once the finger touches the silicone rubber again.

A proof‐of‐concept demonstration was conducted to showcase the functionality of the underwater TENG. Interestingly, the rubber glove could initiate the triboelectric effect by tapping the water surface where the TENG was positioned (Movie S4, Supporting Information). When the rubber glove was lifted off the water surface, only a noise signal was generated (Figure 6d; Figure S46a, Supporting Information). However, as soon as the rubber glove came into contact with the water surface again, a triboelectric signal emerged (Figure 6e). Surprisingly, the output voltage produced by the underwater TENG was even higher than that generated by tapping the TENG in air (Figure 6f; Figure S46b and Movie S5, Supporting Information). It was worth noting that, compared to the operation process of the TENG in air, a slight fluctuation of the noise signal in the output voltage was observed during the operation of the TENG underwater. This could be attributed to the effect of water wave flow on the output performance. Furthermore, it is important to highlight that the triboelectric effect was not limited to the utilization of a rubber glove, any triboelectric material could be employed to initiate the underwater TENG. As demonstrated in Figure 6g and h, steel and copper could also function as triboelectric materials to enable the proper operation of the TENG underwater. Additionally, common plastics also served as triboelectric materials as well. The corresponding results summarized in Figure 6i and Figure S47 (Supporting Information) indicated that different triboelectric materials led to variations in the output performance of the TENG. These variations were attributed to the inherent electronegativity and triboelectric charge density of the materials.^[^ ^43^ ^]^

For comparison, we also conducted a systematic study on the output performance of the PS hydrogel‐based TENG in air. The results, shown in Figure S48 (Supporting Information), demonstrated the satisfactory electrical output performance of the TENG, including an open‐circuit voltage of 60 V, a short‐circuit current of 1000 nA, and a charge transfer amount of 20 nC at an operating frequency of 3 Hz. We observed that altering the operating frequency led to variations in the TENG performance. Specifically, the short‐circuit current increased with the increment of the frequency, while the open‐circuit voltage and the charge transfer amount remained relatively unchanged. This could be attributed to the shortened contacting time between the triboelectric materials at higher frequencies, resulting in a higher charge flow rate and an enhanced current output. Furthermore, the choice of different triboelectric materials also affected the output performance of the TENG in air, as shown in Figure S49 (Supporting Information). These findings further demonstrated the versatility and potential of the PS hydrogel‐based TENG in various settings, including underwater applications and air‐based energy harvesting, showcasing its potential for practical use and promising future developments in the field.

## 3 Conclusion

In summary, our study presented the development of a hydrogel that exhibited exceptional anti‐swelling properties and robust underwater adhesion using the water‐induced phase separation technique. By strategically leveraging the distinct affinity of polymer chains to water, we achieved phase separation within the hydrogel network, leading to the formation of hydrophobic and hydrophilic polymer phases. This unique architecture contributed to the remarkable anti‐swelling behavior of the hydrogel, which remained mechanically intact even after prolonged exposure to water. The presence of rich hydrophobic polymer phases not only facilitated the elimination of the hydration layer at the gel‐substrate interface but also promoted strong interactions between the adhesion functional groups and substrates, resulting in superior underwater adhesion properties of the phase‐separated hydrogel. Additionally, the phase‐separated structure enhanced the mechanical strength and energy dissipation capabilities of the hydrogel, further augmenting its adhesive performance. To expand the practical applications of the hydrogel, MWCNT‐COOH was incorporated, which exhibited a negligible effect on the anti‐swelling and adhesion properties of the hydrogel and resulted in a stable and constant conductivity underwater. The conductive PS hydrogel demonstrated high efficiency as a crucial component for various underwater applications, including underwater sensing, communication, and energy harvesting. Our findings highlighted the water‐induced phase separation approach as a promising strategy for developing durable underwater adhesives and advancing underwater soft electronics. The combination of anti‐swelling properties, robust adhesion, and conductivity positioned our hydrogel system a significant contender for underwater technology applications.

## 4 Experimental Section

### Materials

Acrylic acid (AAc), 2‐ethylhexyl acrylate (EHA), 2‐Methoxyethyl acrylate (MEA), 2,2,2‐Trifluoroethyl methacrylate (TFEA), 2,2,3,4,4,4‐hexafluorobutyl 2‐methylprop‐2‐enoate (STAB), tannic acid (TA), carboxylic multiwalled carbon nanotube (MWCNT‐COOH, length of 10–30 µm), 5‐Carboxyfluorescein (5‐FAM), and bromocresol green were provided by Aladdin Biochemical Technology Co., Ltd (Shanghai, China). Potassium persulfate (KPS) and Congo Red were purchased from Bodi Chemical Reagent Co., Ltd (Tianjin, China). Dimethyl sulfoxide (DMSO) was supplied by Paini Chemical Reagent Co., Ltd (Zhengzhou, China). N, N’‐methylene diacrylamide (MBAA), hexane, toluene, and dodecane were purchased from Kelong Chemical Reagent Factory (Chengdu, China). Deionized water was supplied by Changzheng Chemical Reagent Co., Ltd (Chengdu, China). All reagents were used as received without further purification. Artificial seawater was prepared by dissolving KCl (7 g L^−1^), MgSO_4_ (3.2 g L^−1^), MgCl_2_ (2.3 g L^−1^), CaCl_2_ (1.15 g L^−1^), and NaCl (26.7 g L^−1^) in deionized water. The acid solutions with pH = 3 and 5 were prepared by diluting hydrochloric acid with deionized water.

### Preparation of Phase‐Separated Hydrogels

The solvent exchange method was employed to fabricate the phase‐separated hydrogels. Initially, organogels were prepared through one‐pot free radical polymerization. As an illustrative example, AAc (0.03 mol), EHA (0.005, 0.01, 0.02, and 0.03 mol), TA (0, 10, 20, and 30 mg), and KPS (0.2 mol/mol% relative to the total monomer quantity) were dissolved in 10 g of DMSO. The precursor solution was stirred and purged with nitrogen, and then injected into a homemade reaction cell for polymerization at 75 °C for 6 h. Subsequently, the organogels were immersed in a large volume of water for solvent exchange. The water was exchanged every 12 h for 3 days to ensure complete removal of DMSO. The resulting phase‐separated hydrogels were denoted as PAE*_x_*/T*_y_*, where *x* (1/6, 1/3, 2/3, and 1) represented the molar ratio of EHA to AAc, and *y* (0, 10, 20, and 30) indicated the mass of TA in the precursor solution. Unless otherwise stated, the PA_3_E_1_/T_20_ hydrogel (PAE_1/3_/T for short) was utilized as the typical sample for tests and characterizations in this work. Changing the hydrophobic monomer (MEA, TFEA, STAB) and preparing the PAM_1/3_/T, PAF_1/3_/T, and PAS_1/3_/T gels by the same preparation method, respectively.

### Preparation of Non‐Phase‐Separated Hydrogels

As control samples, non‐phase‐separated hydrogels were also prepared without the addition of EHA through free radical polymerization. Specifically, AAc (0.03 mol), TA (20 mg), KPS (0.2 mol/mol% relative to AAc) and MBAA (0.1 mol/mol% relative to AAc) were dissolved in 10 g of deionized water. The precursor solution was stirred and purged with nitrogen for 15 min, followed by injection into the reaction cell for polymerization at 75 °C for 6 h. Subsequently, the non‐phase‐separated hydrogels, named PAE_0_/T, were obtained.

### Preparation of Conductive Phase‐Separated Hydrogels

To impart electrical conductivity to the phase‐separated hydrogels, MWCNT‐COOH was incorporated. Initially, a certain amount of MWCNT‐COOH (0.3, 0.5, 0.7, and 0.9 wt.% of the total mass of the precursor solution) was dispersed in 10 g of DMSO using ultrasonic vibration for 1 h. Subsequently, AAc (0.03 mol), EHA (0.005, 0.01, 0.02, and 0.03 mol), TA (0, 10, 20, and 30 mg), and KPS (0.2 mol/mol% relative to the sum of monomers) were dissolved in the precursor solution. The solution was then stirred, bubbled with nitrogen, and injected into the reaction cell for polymerization at 75 °C for 6 h. The resulting organogels were subjected to solvent exchange by immersion in water. The final conductive phase‐separated hydrogels were denoted as PAE*_x_*/T*_y_*/C_z_, where *x* (1/6, 1/3, 2/3, and 1) represented the molar ratio of EHA to AAc, *y* (0, 10, 20, and 30) indicated the mass of TA in the precursor solution, and *z* represented the mass fraction of MWCNT‐COOH (0.3, 0.5, 0.7, and 0.9) in the precursor solution. The composition contents of all hydrogels used in this study were summarized in Table S2 (Supporting Information).

### Fabrication of the Triboelectric Nanogenerator (TENG)

The TENG was constructed by assembling a conductive phase‐separated hydrogel (20 × 20 × 1.5 mm^3^) between two pieces of silicone rubber (30 × 30 × 0.5 mm^3^). Copper foil was connected to the hydrogel as an external electrode.

### Statistical Analysis

The data with an error bar were represented as mean ± standard deviation calculated on a minimum of three independent samples using the Origin Software.

## Conflict of Interest

The authors declare no conflict of interest.

## Supporting information

Supporting Information

Supplemental Movie 1

Supplemental Movie 2

Supplemental Movie 3

Supplemental Movie 4

Supplemental Movie 5

## Data Availability

The data that support the findings of this study are available from the corresponding author upon reasonable request.
