# Supplementary material for: Water‐Induced Phase Separation for Anti‐Swelling Hydrogel Adhesives in Underwater Soft Electronics
Source: Adv Sci (Weinh). 2023 Sep 26;10(32):2304780. doi: 10.1002/advs.202304780 (PMC10646223; doi:10.1002/advs.202304780)
Supplement: Supplementary file 1 — Supporting Information [file ADVS-10-2304780-s003.pdf]

ADVANCED SCIENCE
Open Access

Supporting Information

for *Adv. Sci.*, DOI 10.1002/advs.202304780

Water-Induced Phase Separation for Anti-Swelling Hydrogel Adhesives in Underwater Soft Electronics

Min Li, Honglang Lu, Menghan Pi, Hui Zhou, Yufei Wang, Bin Yan, Wei Cui and Rong Ran**

Supporting Information

Water-Induced Phase Separation for Anti-Swelling Hydrogel Adhesives in Underwater Soft Electronics

Min Li, *Honglang Lu, Menghan Pi, Hui Zhou, Yufei Wang, Bin Yan, Wei Cui,** and *Rong Ran**

College of Polymer Science and Engineering, Sichuan University, Chengdu 610065, China.

*Corresponding authors:

(W.C.) cuiwei@scu.edu.cn;

(R.R.) ranrong@scu.edu.cn.

The SI file includes:

1. Supplementary Methods
2. Supplementary Figure S1-S49
3. Supplementary Table S1-S2
4. Supplementary Movie Captions
5. Supplementary Reference

Supplementary Methods

***Characterization Techniques**:* Ultraviolet-visible (UV) spectroscopy (UV-3600, Japan) was utilized to measure the transmittance of gels within the wavelength range of 400-800 nm. The absorption spectrum of liquids was measured in the wavelength range of 300-800 nm. Attenuated total reflection Fourier-transform infrared (ATR FT-IR) spectrometry (Nicolet IS50, USA) was used to characterize the hydrogel composition. To minimize water interference in the characterization results, the gel samples were equilibrated in deuterium oxide for 12 hours before measurement. For visualizing the gel microstructure, samples were frozen in liquid

nitrogen, fractured, and freeze-dried. Scanning electron microscopy (SEM) (Apreo S HiVoc, USA) was used to observe the cross-sectional surface of the samples. The average pore diameter of the gels was analyzed using Image J software. X-ray Diffraction (XRD) patterns were obtained using X-ray diffraction (Ultima IV, Japan) with nickel-filtered Cu-Kα (λ = 1.541 Å) radiation. The water contact angle at room temperature was measured using a DSA25 contact angle measuring device (KRUSS, Germany). Confocal laser scanning microscopy (Nikon N-SIM, Japan) was employed to image the phase-separated structures of the hydrogels, with 5-Carboxyfluorescein added to label the carboxyl group in the precursor solution. Nuclear Magnetic Resonance (NMR) Spectrometer (AV III HD 400 MHz, Germany) was used to confirm the complete removal of DMSO from the hydrogel after solvent exchange. For NMR measurement, the hydrogel was equilibrated in deuterium oxide for 12 hours to extract the internal solution. The Small-angle X-ray Scattering (SAXS) System (Xeuss 2.0, France) provided structural data of NPS and PS gels, with X-ray energy of 50 keV, wavelength of X-ray as 1.54189 Å, and a sample-to-detector distance of 2841.36 mm. 2D SAXS patterns were recorded using the PILATUS3 300K detector and analyzed with Foxtrot-Academic-Edition software. The hydrogel-pigskin interface was observed using an ultra-depth three-dimensional microscope (VHX-1000C, Japan) in both air and underwater conditions.

***Mechanical Tests**:* The hydrogels were initially cut into a dumbbell shape measuring 20×4×2 mm^3 to conduct the uniaxial tensile test using an Instron universal test instrument (Model 5576, USA) with a 500 N load cell at room temperature. The tensile velocity was set at a constant rate of 100 mm min^{-1}, corresponding to a strain rate of 0.083 s^{-1}. The tensile strain was determined as the change in length relative to the initial length of the sample. Young's modulus was calculated from the initial linear range of the stress-strain curve. For the single loading-unloading tensile test, the samples were stretched to a fixed strain and then unloaded at the same velocity of 100 mm min^{-1}. To investigate the behavior at different strains, the samples underwent loading-unloading cycles at 100 mm min^{-1} under various strains ranging from 50% to 800%. To assess the cyclic loading-unloading performance, the samples were subjected to continuous stretching at a fixed strain with a velocity of 100 mm min^{-1} for 10 cycles without any resting time. The dissipated energy, or hysteresis, was determined by integrating the area between the loading and unloading curves.

***Rheological Measurements**:* Rheological measurements of the hydrogels were conducted using a rheometer (MCR302, Austria) equipped with a parallel plate having a diameter of 8 mm. A frequency sweep was performed over the range of 1-100 rad/s at a fixed strain of 1%. All rheological measurements were carried out at a temperature of 25 ± 0.1 °C, controlled by a Peltier plate.

***Adhesion Performance Evaluated by Lap-Shear Tests**:* The hydrogel samples were cut into rectangular shapes measuring 25×15×1.5 mm³ for lap-shear tests conducted in both air and underwater conditions. During the test, the hydrogel was gently placed between two substrates, ensuring proper sandwiching. A weight of 100 g was applied to the top of the sandwich for 30 minutes to ensure full contact between the gel and the substrates before testing. The testing velocity was set at 50 mm/min, and the adhesion strength was defined as the maximum tensile force (Fmax) per nominal contact area, represented as $\tau_s = F_{max}/wl$,[1] where w and l are the width and length of the contact area, respectively.

180-degree peeling tests for assessing adhesion energy: Hydrogel samples were cut into rectangular shapes with a dimension of 25×65×1.5 mm³, tailored for underwater testing conditions. The unattached ends of both the hydrogel and the substrate were clamped by the tensile tester and displaced at a testing velocity of 50 mm/min. Adhesion energy was calculated as twice the peeling force divided by the gel width when a force plateau is reached.

Swelling Tests: The swelling ratio was employed to evaluate the anti-swelling capability of the hydrogel. The gel samples were immersed in solvents that were 50 times the mass of the gel at room temperature, and the mass of the gel was measured daily. The swelling ratio (SR) was calculated using the following equation:

$$\mathrm{SR} = \frac{m_s}{m_0}\,(\mathrm{g/g})$$

where m_0 represents the initial weight of the hydrogel sample, and m_s represents the weight of the swollen hydrogel in different solvents.

In addition to quantifying the swelling ratio based on weight change, we also measured the volume-swelling ratio of the hydrogels. The gel samples were initially cut into disks with an initial diameter of 20 mm and then immersed in deionized water until reaching equilibrium. The diameter of the swollen gel was measured, and the volume-swelling ratio (Q) was determined assuming isotropic swelling using the following equation:

$$Q = \left({}^{d}/_{d_0} \right)^3$$

where d_0 represents the initial diameter of the gel and d represents the diameter of the swollen gel.

Conductivity Measurements: An electrochemical workstation (CHI 760E, China) was utilized to measure the electrochemical impedance spectra (EIS) of the gel samples with a frequency range of 0.01-100000 Hz and a potential amplitude of 5 mV under open-circuit conditions. The conductivity (σ) was calculated using the formula:

$$\sigma = \frac{d}{R \times A}$$

where d represents the distance between adjacent electrodes, R and A represent the resistance and cross-sectional area of the gel sample, respectively.

The resistance (R) of the hydrogels was measured using a Keithley 2601B system source meter. The relative resistance change ($\Delta R/R_0$) was calculated using the equation:

$$\frac{\triangle R}{R_0} = \frac{R_r - R_0}{R_0} \times 100\%$$

where R_r represents the real-time resistance of the hydrogel during deformation and R_0 is the resistance of the hydrogel at the initial state. The gauge factor (GF) was defined as $\mathrm{GF} = \left(\frac{\triangle R}{R_0}\right) / \varepsilon$, where ε represents the applied strain.

The Output Performance of TENG: To evaluate the output performance of the triboelectric nanogenerator (TENG), it was driven by a linear motor, and the output performance was measured accordingly. The output performance at different operating frequencies was obtained by controlling the step speed of the linear motor. The output performance of the TENG was measured using a Keithley 6517 electrometer.

Supplementary Figures

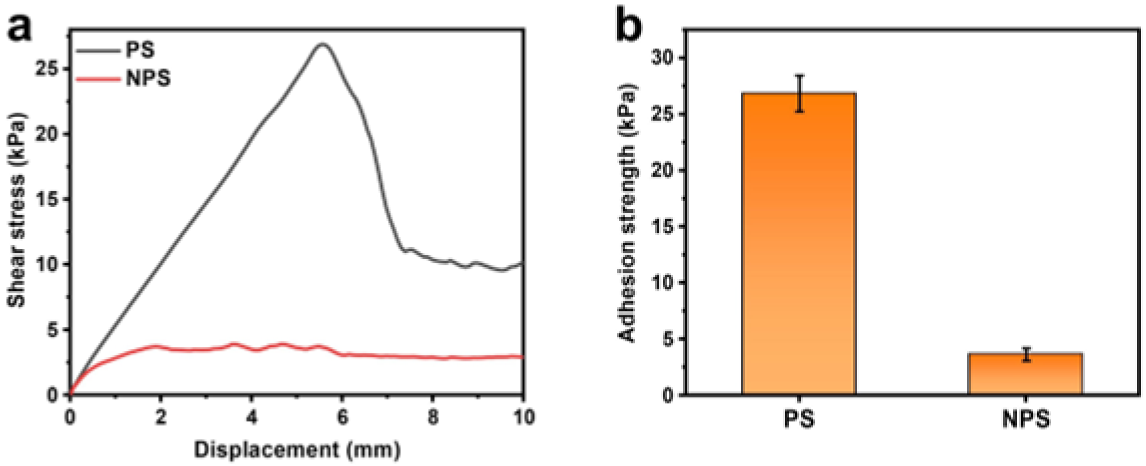

Figure S1. Contrasting underwater adhesion performance between PS and NPS hydrogels. (a) Shear adhesion stress *versus* displacement for the PS and NPS hydrogels on polypropylene underwater. (b) Summary of the adhesion strength of the PS and NPS hydrogels to polypropylene underwater.

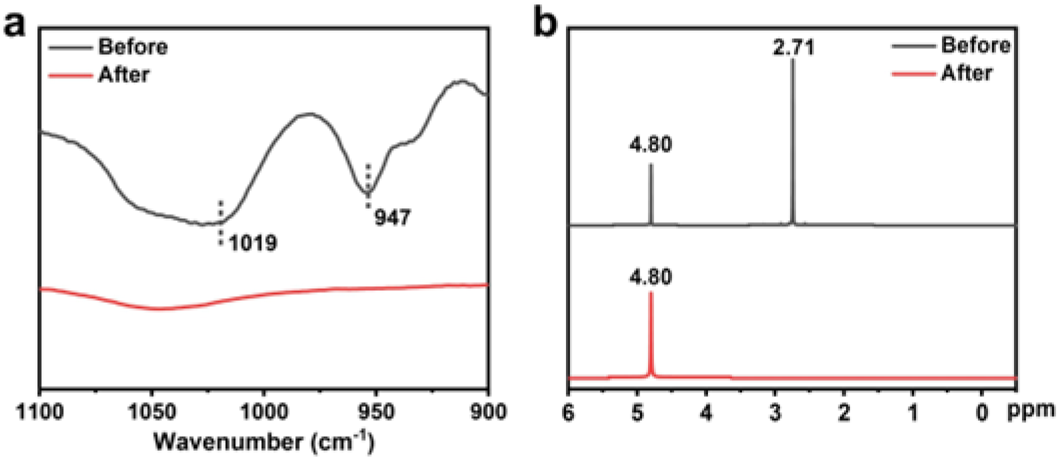

Figure S2. Characterization of the $PAE_{1/3}$/T gel before and after solvent exchange. (a) ATR FT-IR spectra showing the comparison between the $PAE_{1/3}$/T gel before and after solvent exchange. (b) 1H NMR spectra illustrating the changes in the solvents inside the $PAE_{1/3}$/T gel before and after solvent exchange.

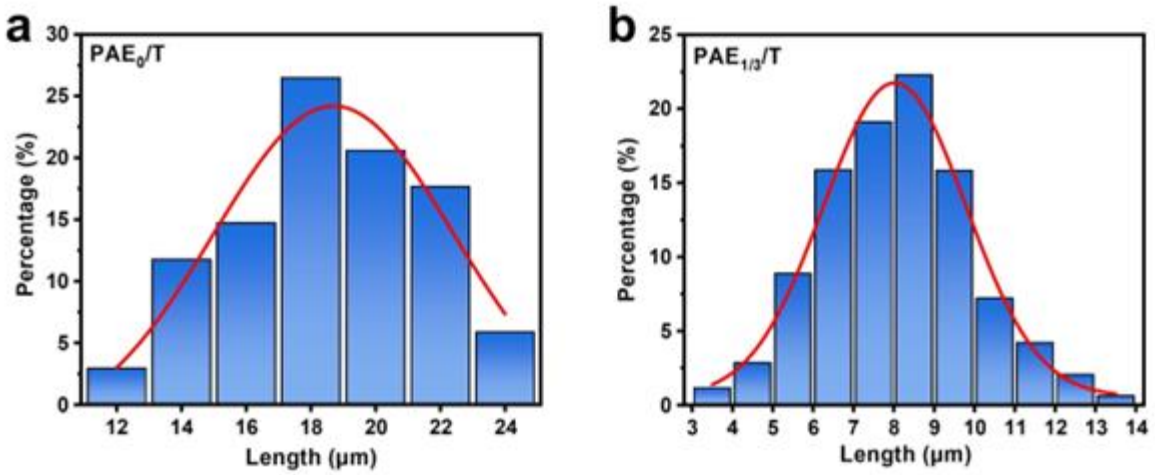

Figure S3. The Pore diameters of the gel distribution histogram. (a) The PAE$_0$/T gel. (b) The PAE$_{1/3}$/T gel.

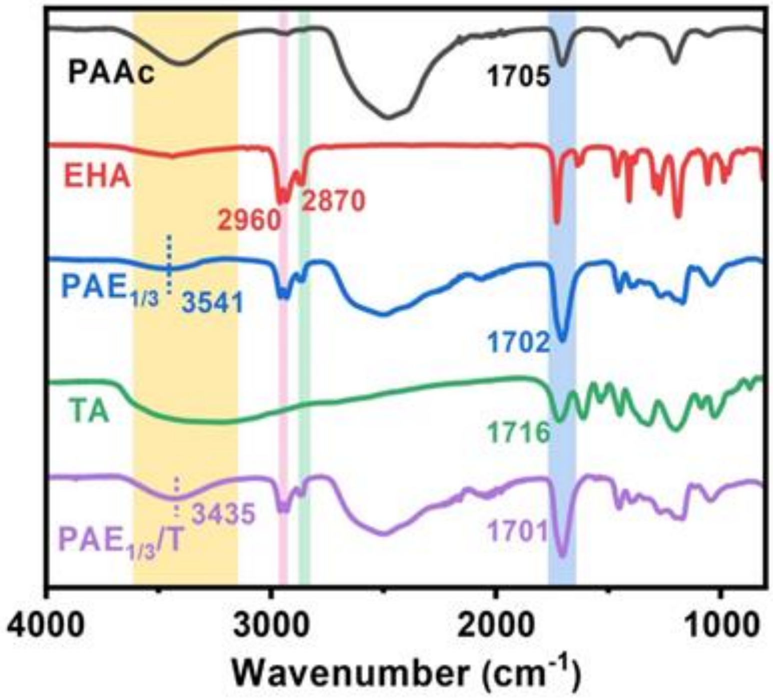

Figure S4. ATR FT-IR spectra of different components comprising the PS gel. A comparison of the spectra of neat PAAc with those of PAE$_{1/3}$ and PAE$_{1/3}$/T samples revealed new characteristic peaks at 2960 cm^{-1} and 2870 cm^{-1}, corresponding to *v*(-CH_3), indicating successful copolymerization of EHA with AAc. Additionally, the characteristic peak corresponding to *v*(-OH) shifted from 3541 cm^{-1} in the PAE$_{1/3}$ sample to 3435 cm^{-1} in the PAE$_{1/3}$/T sample, suggesting hydrogen bonding between TA and PAE polymer chains.

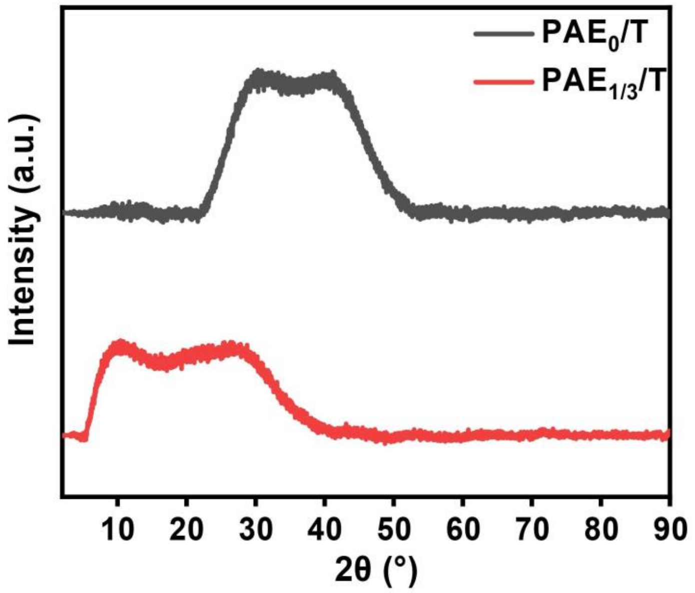

Figure S5. XRD profiles of the NPS gel (PAE_0/T) and the PS gel ($PAE_{1/3}$/T).

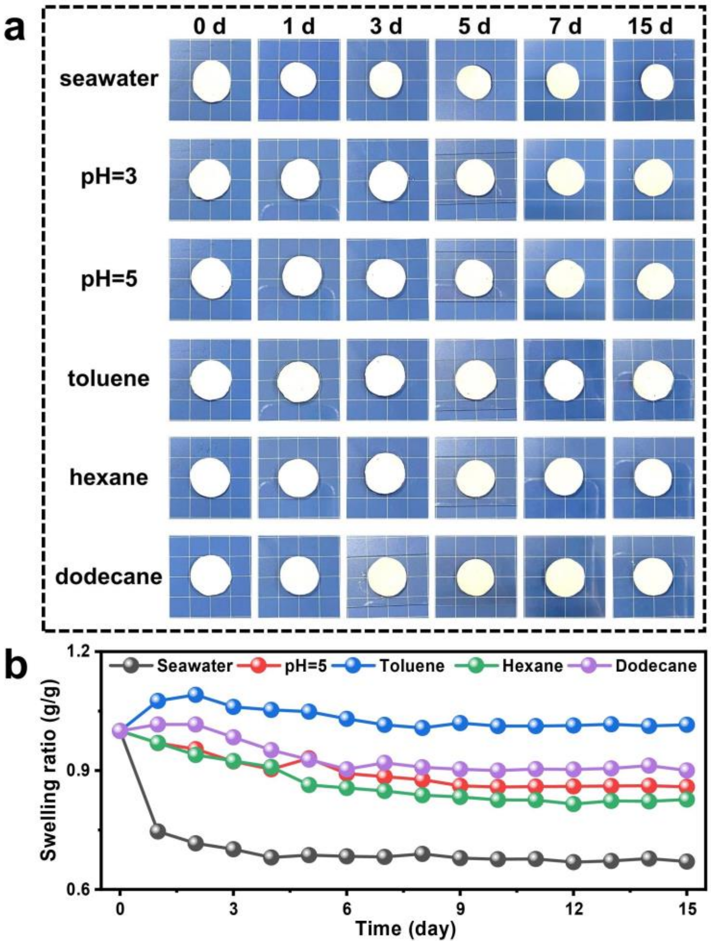

Figure S6. Strong swelling resistance of the PS $PAE_{1/3}$/T hydrogel to a variety of solvents. (a) Photographs illustrating the negligible size change of the $PAE_{1/3}$/T gel soaked in different solutions for up to 15 days. The background grid size in all photographs is 10 mm. (b) Swelling ratio of the $PAE_{1/3}$/T gel during the immersion in different solutions for 15 days.

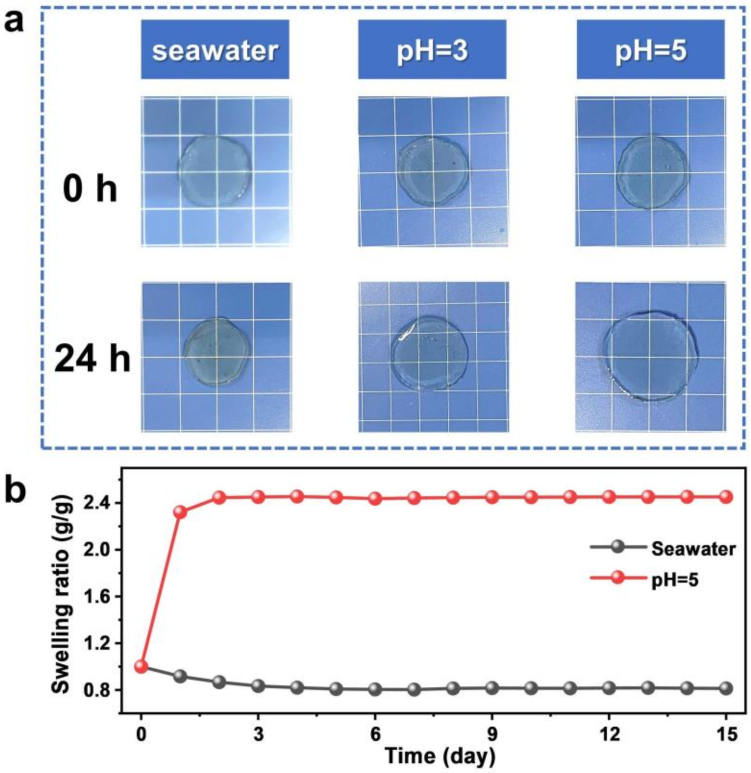

Figure S7. Weak swelling resistance of the NPS PAE0/T hydrogel to solvents. (a) Photographs of the PAE_0/T gel before and after being soaked in different solvents. (b) The swelling ratio of the PAE_0/T gels soaked in different solutions for different days. The background grid size in all photographs is 10 mm.

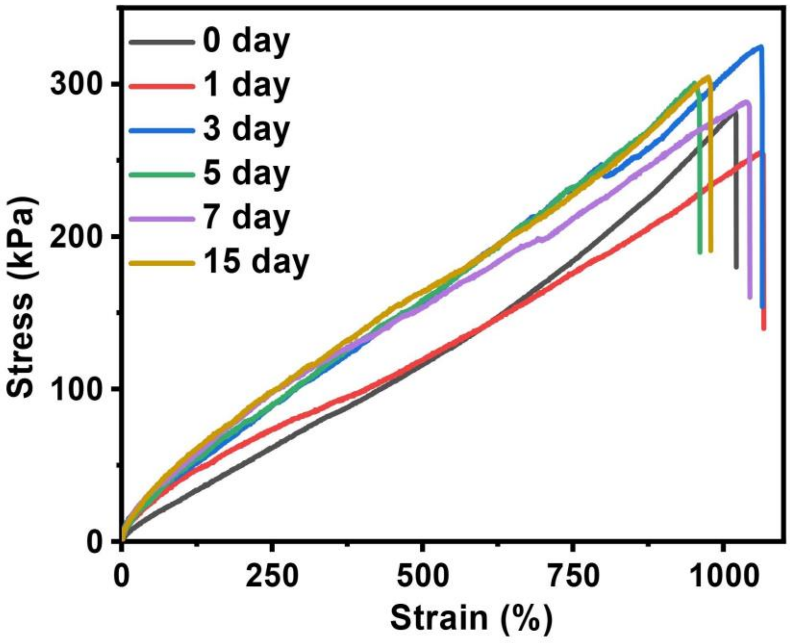

Figure S8. Tensile stress-strain curves of the $PAE_{1/3}$/T gel after immersion in artificial seawater for various durations.

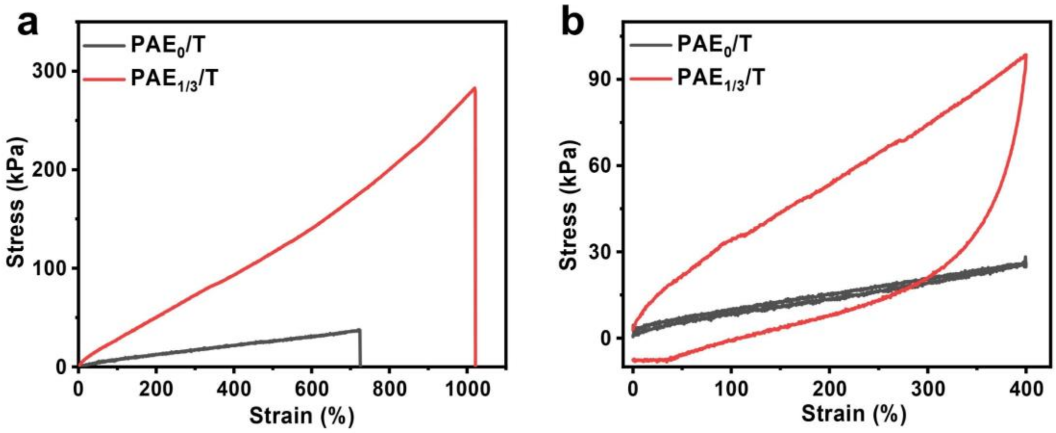

Figure S9. Comparison of mechanical properties between the PS and NPS hydrogel. (a) Tensile stress-strain curves of the NPS PAE_0/T and PS $PAE_{1/3}$/T gels, respectively. (b) Tensile loading-unloading curves of the NPS PAE_0/T and PS $PAE_{1/3}$/T gels at a fixed strain of 400%, respectively.

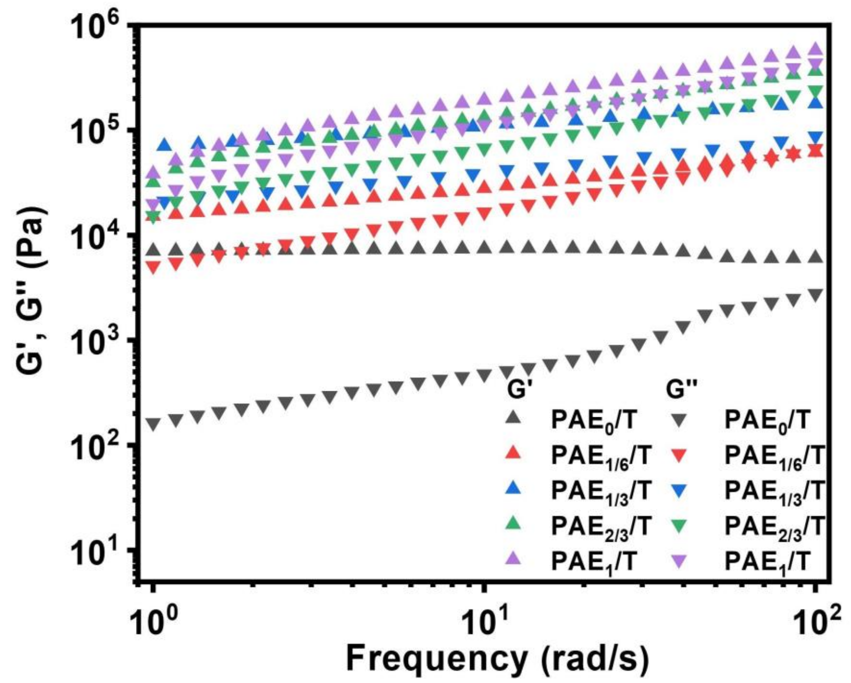

Figure S10. Storage modulus and loss modulus of PAE_x/T gels with different PEHA contents in a frequency range of 1 to 100 rad s^{-1}. The hydrogels containing PEHA exhibited a frequency-dependent storage modulus, indicating a viscoelastic nature.

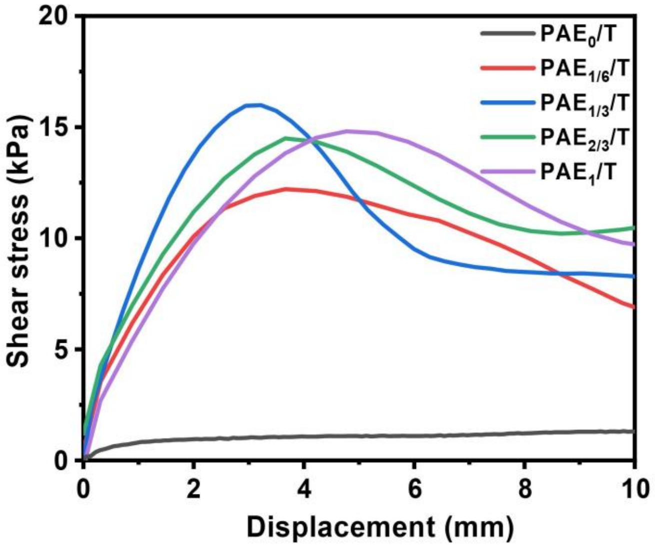

Figure S11. Effect of PEHA content on the shear adhesion strength of PAE_x/T hydrogels to glass underwater.

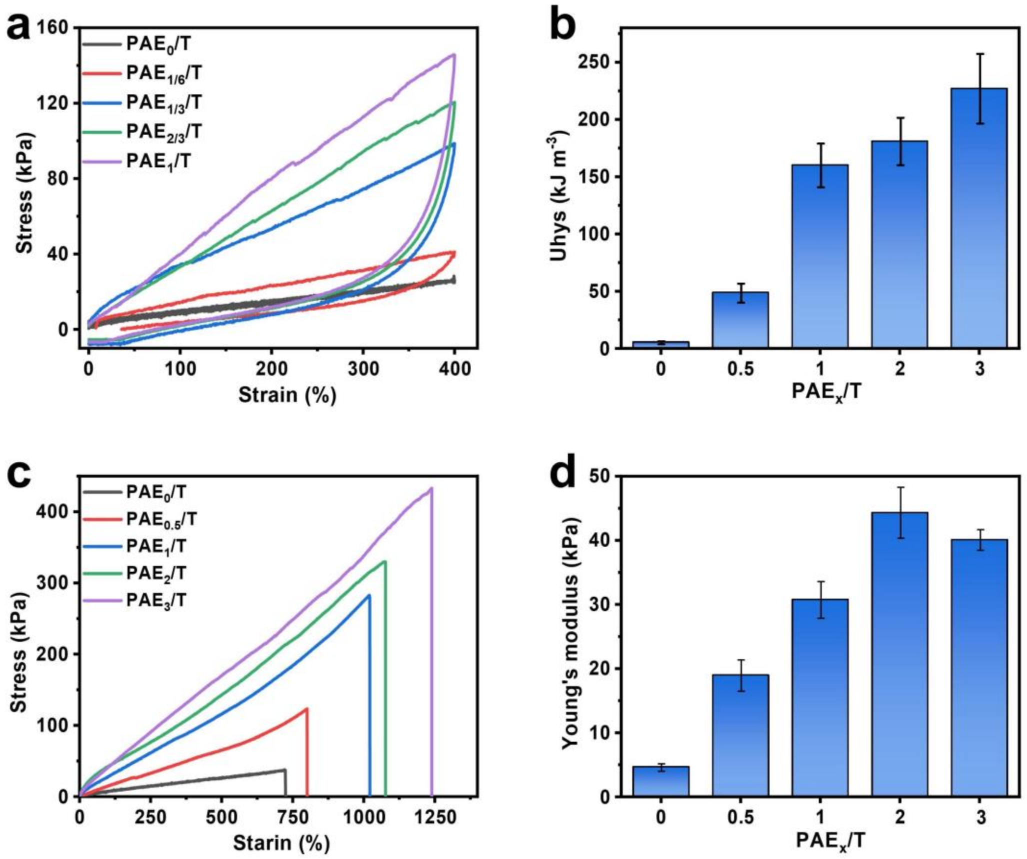

Figure S12. Effect of PEHA content on the energy dissipation capability and tensile behavior of the hydrogel. (a) Tensile loading-unloading curves of PAE_x/T gels with varying PEHA contents at a fixed strain of 400%. (b) Summarized hysteresis (U_{hys}) values for all PAE_x/T hydrogels. (c) Tensile stress-strain curves of PAE_x/T gels with different PEHA contents. (d) Summary of Young's modulus for all PAE_x hydrogels.

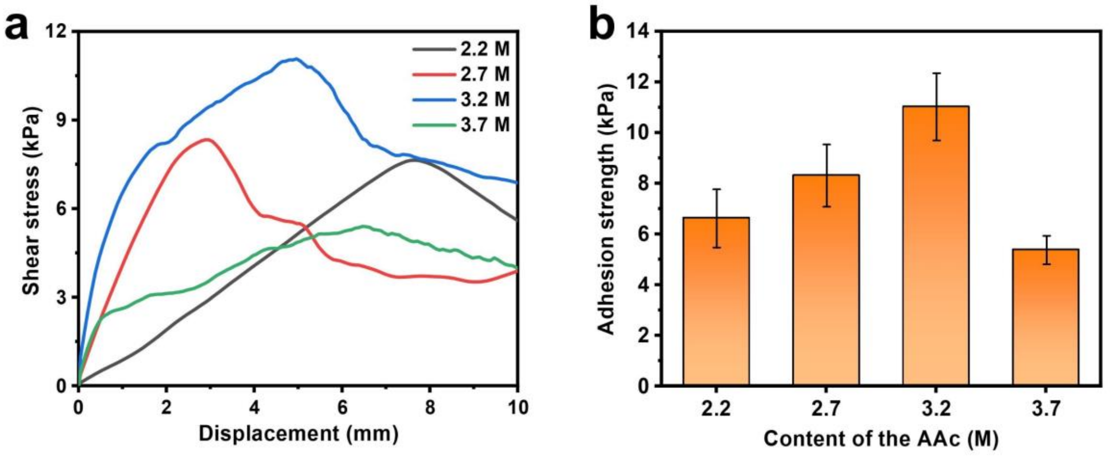

Figure S13. Adhesion performance of the PAE/T hydrogel to pigskin with varied hydrophilic monomer concentrations. (a) Shear adhesion stress *versus* displacement for the PAE/T hydrogel on pigskin with different hydrophilic monomer contents. (b) Summarized adhesion strength data for the PAE/T hydrogel to pigskin prepared with different hydrophilic monomer contents.

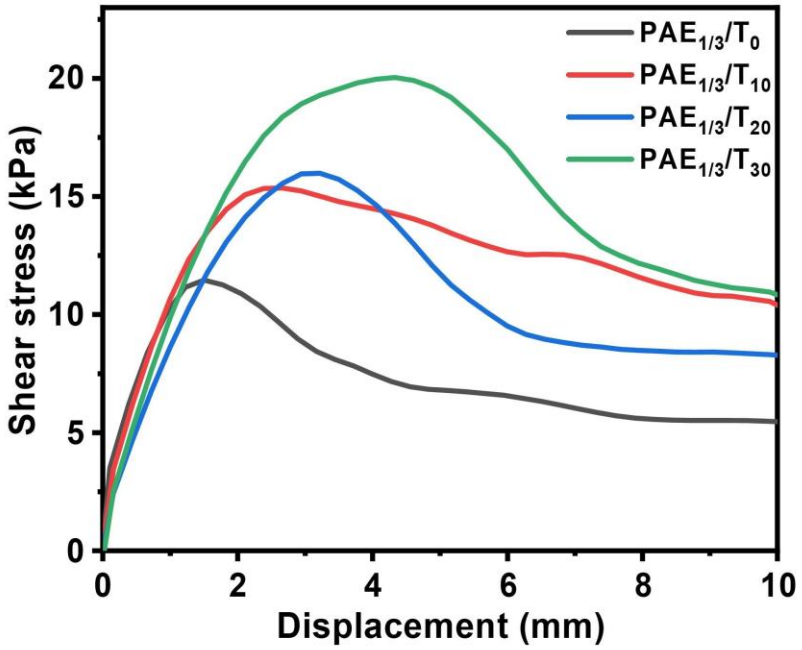

Figure S14. Effect of TA content on the shear adhesion strength of $PAE_{1/3}/T_y$ hydrogels to glass underwater.

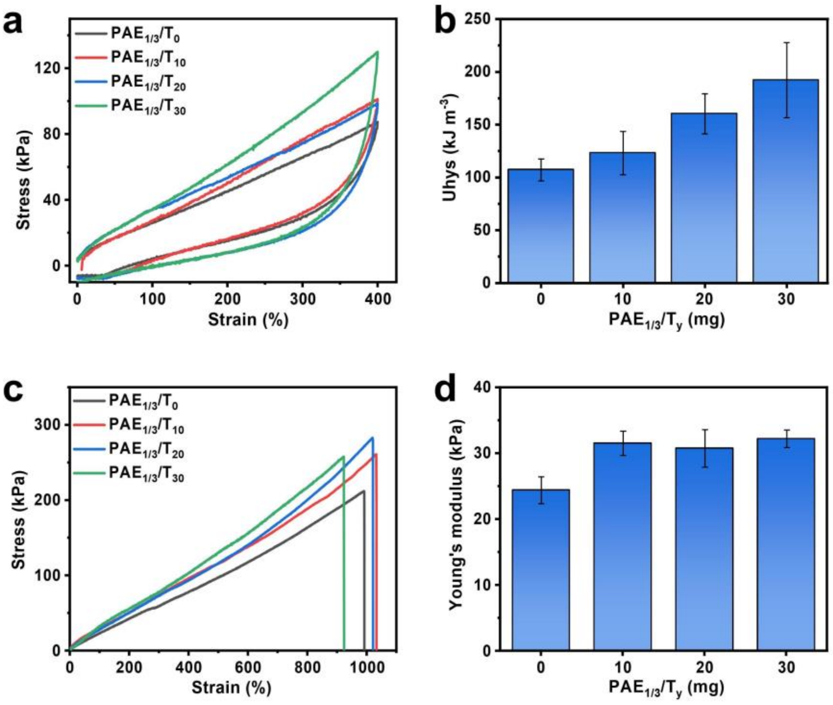

Figure S15. Effect of TA content on the energy dissipation capability and tensile behavior of the hydrogel. (a) Tensile loading-unloading curves of $PAE_{1/3}/T_y$ gels with different TA contents at a fixed strain of 400%. (b) Summarized hysteresis (U_{hys}) values of all $PAE_{1/3}/T_y$ hydrogels. (c) Tensile stress-strain curves of $PAE_{1/3}/T_y$ gels with different TA contents. (d) Summary of Young's modulus for all $PAE_{1/3}/T_y$ hydrogels.

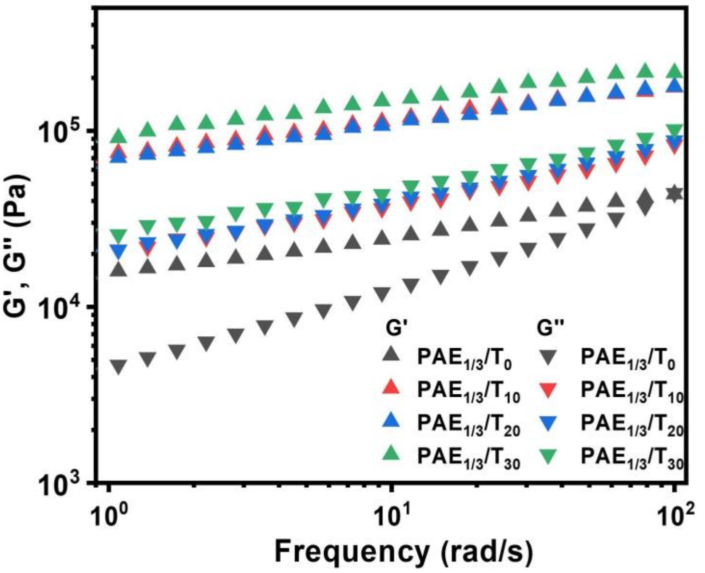

Figure S16. Storage modulus and loss modulus of $PAE_{1/3}/T_y$ gels with different TA contents in a frequency range of 1 to 100 rad s^{-1}.

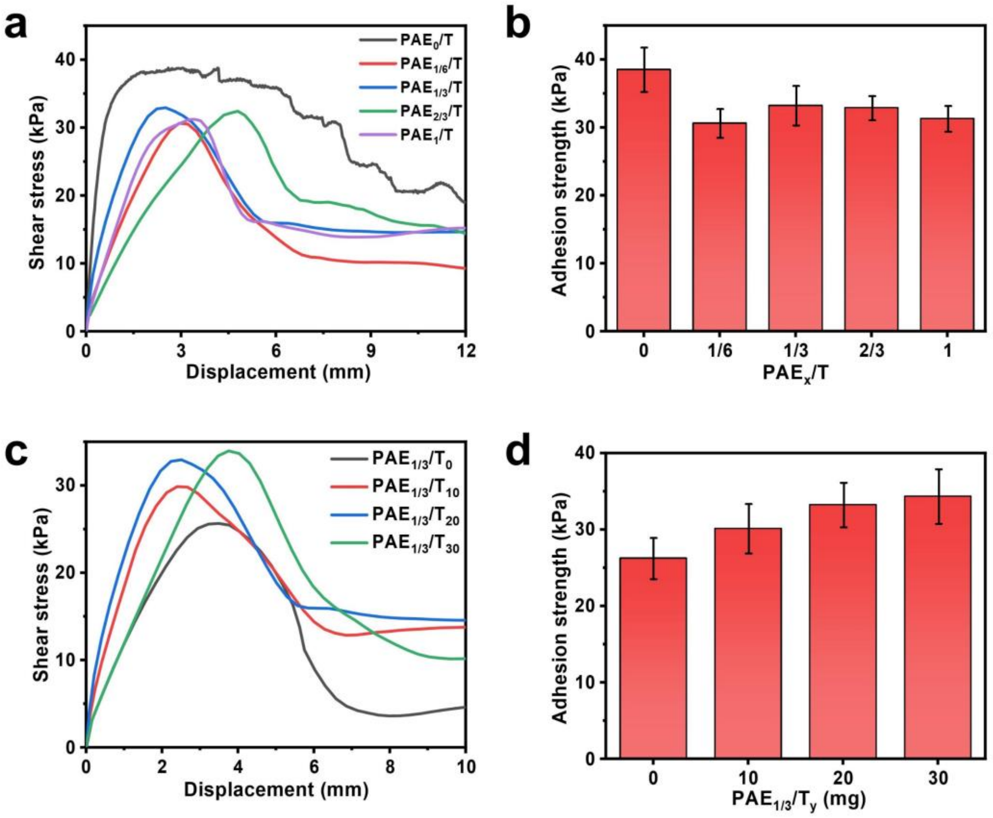

Figure S17. Adhesion performance of PAE/T hydrogels in air. (a) Shear adhesion stress *versus* displacement of PAE_x/T gels with different PEHA contents to glass in air. (b) Summarized adhesion strength of PAE_x/T gels with different PEHA contents to glass in air. (c) Shear adhesion stress *versus* displacement of $PAE_{1/3}/T_y$ gels with different TA contents to glass in air. (d) The adhesion strength of $PAE_{1/3}/T_y$ gels with different TA contents to glass in air.

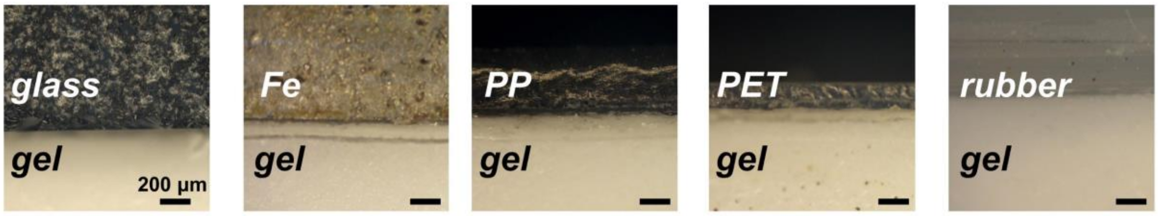

Figure S18. Microscope images of the gel-substrate interface in underwater conditions.

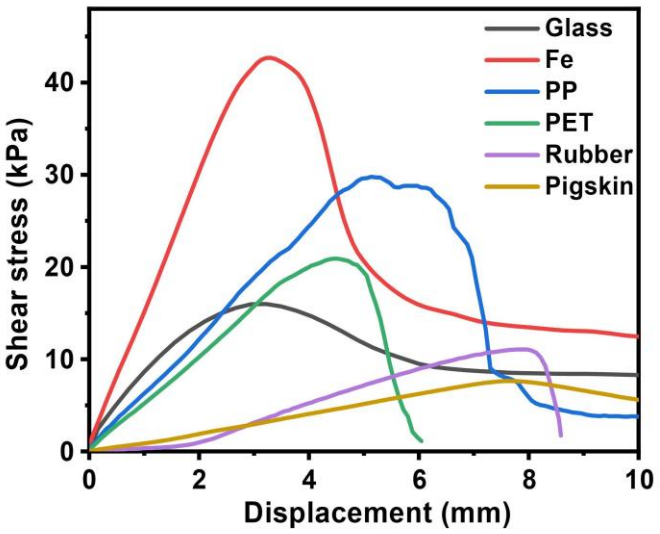

Figure S19. Shear adhesion stress *versus* displacement of the $PAE_{1/3}$/T hydrogel to different substrates underwater.

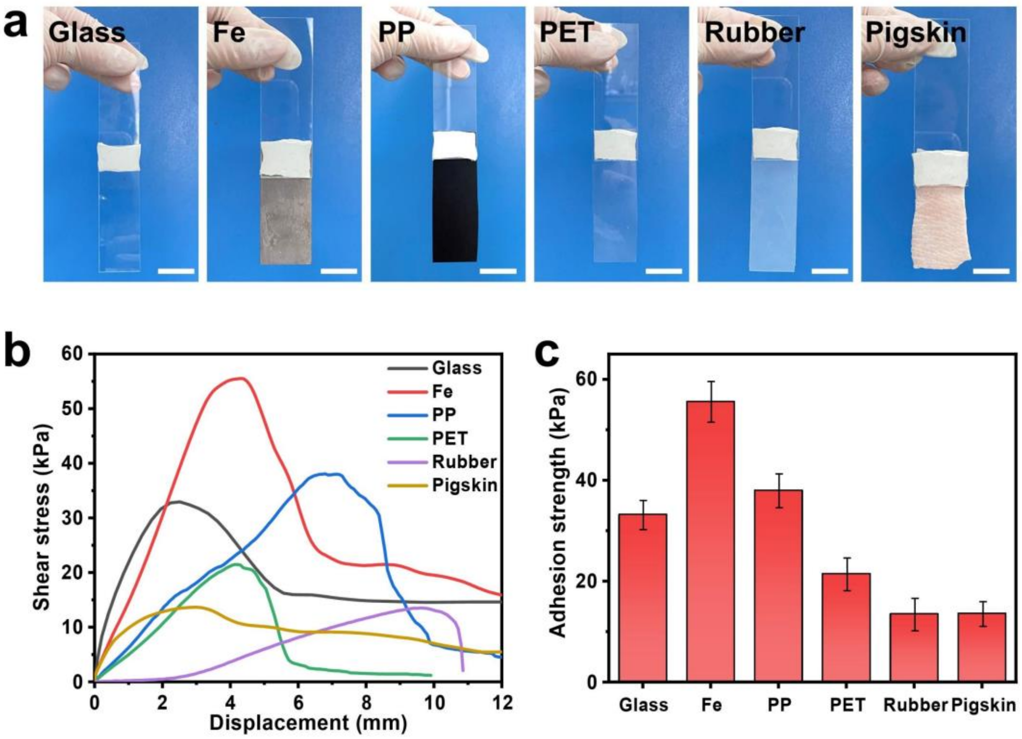

Figure S20. Universal adhesion of the $PAE_{1/3}$/T hydrogel in air. (a) Photographs showing the universal adhesion of the $PAE_{1/3}$/T gel to different substrates in air. The scale bars represent 2 cm. (b) Shear adhesion stress *versus* displacement of the $PAE_{1/3}$/T hydrogel to different substrates in air. (c) The adhesion strength of the $PAE_{1/3}$/T hydrogel to different substrates in air.

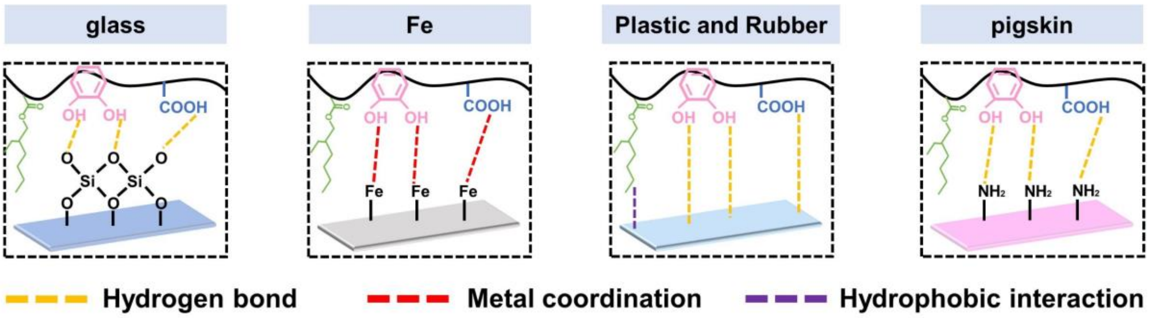

Figure S21. Schematic illustration depicting the molecular interactions between the hydrogel and various substrates.

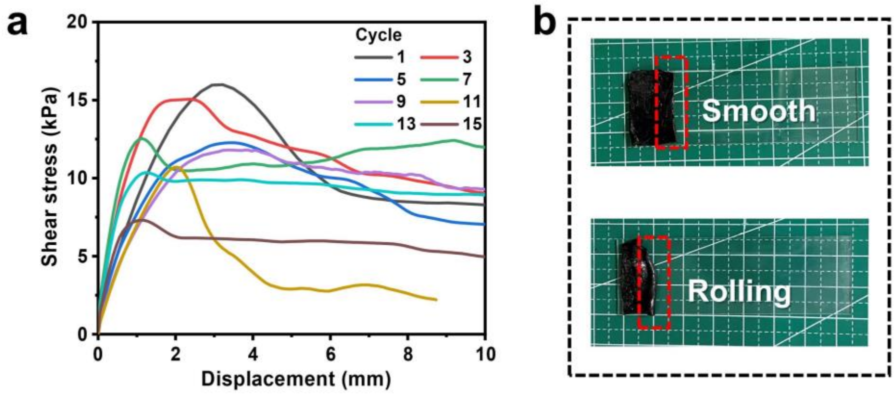

Figure S22. Repeatability of the adhesion of the $PAE_{1/3}$/T hydrogel underwater. (a) Shear adhesion stress *versus* displacement of the $PAE_{1/3}$/T gel after different repeating cycles underwater. (b) Photographs showing the surface appearance of the $PAE_{1/3}$/T gel before and after repeated adhesion. The background grid size is 10 mm.

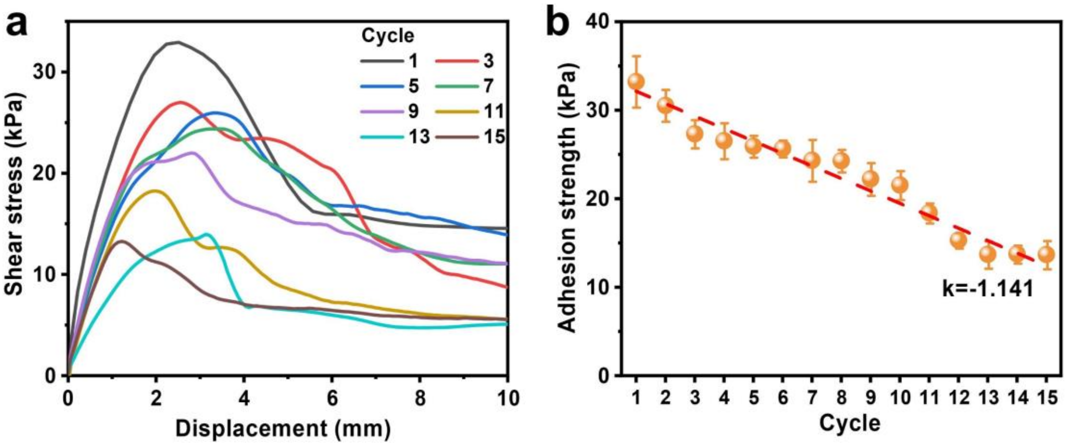

Figure S23. Repeatability of the adhesion of the $PAE_{1/3}$/T hydrogel in air. (a) Shear adhesion stress *versus* displacement of the $PAE_{1/3}$/T gel after different repeating cycles in air. (b) The corresponding shear adhesion strength of the $PAE_{1/3}$/T gel after different repeating cycles in air.

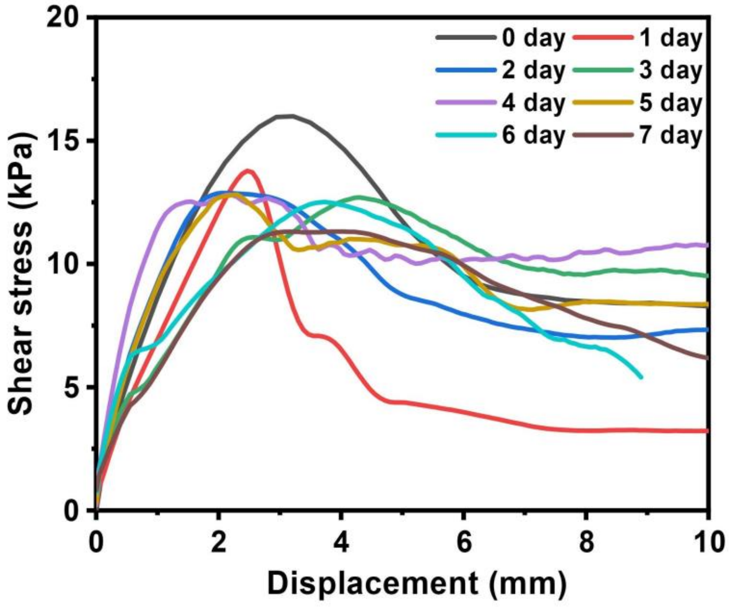

Figure S24. Shear adhesion stress *versus* displacement of the $PAE_{1/3}$/T gel after being soaked in water for different periods.

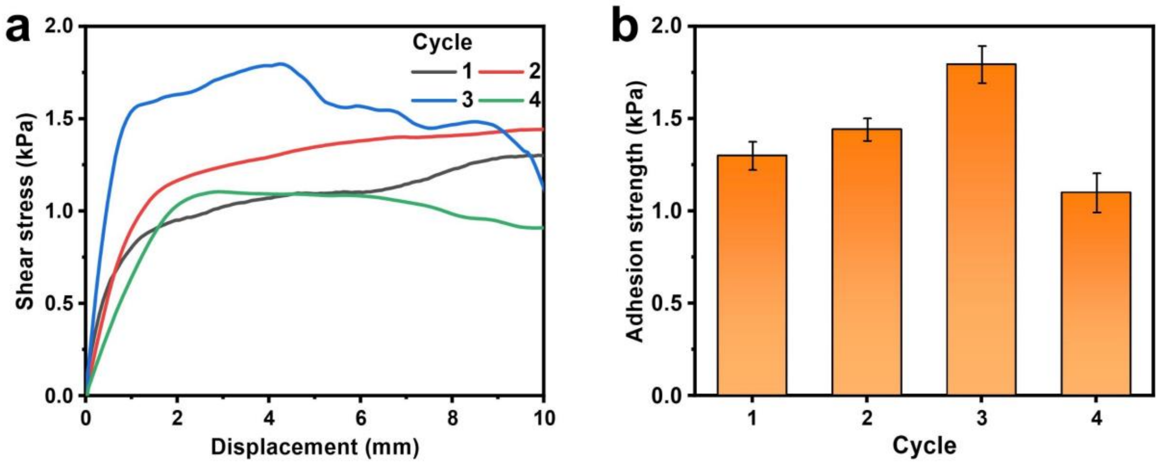

Figure S25. Repeatability of the adhesion of the PAE_0/T hydrogel underwater. (a) Shear adhesion stress *versus* displacement of the PAE_0/T gel after different repeating cycles underwater. (b) The corresponding shear adhesion strength of the PAE_0/T gel after different repeating cycles underwater.

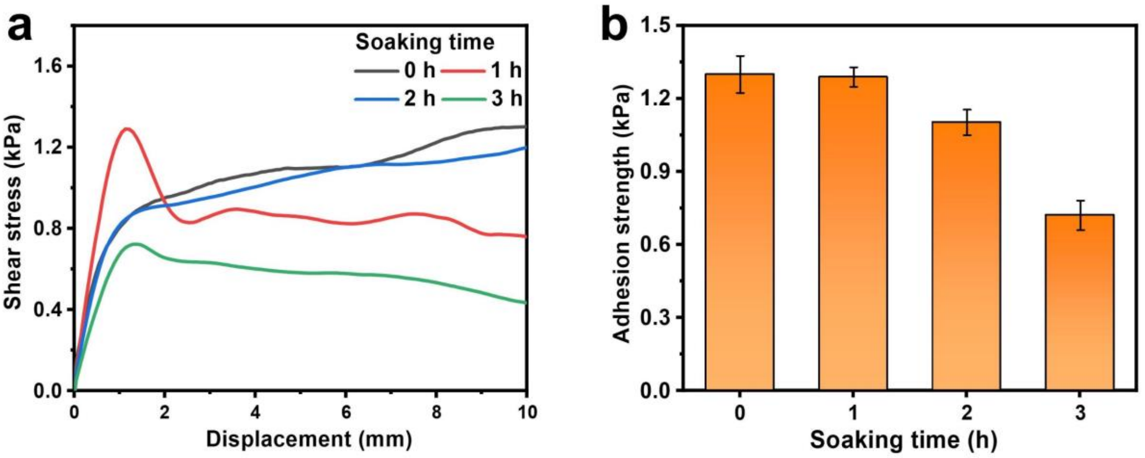

Figure S26. Adhesion performance of the PAE_0/T hydrogel after being soaked in water for different periods. (a) Shear adhesion stress *versus* displacement of the PAE_0/T hydrogel after being soaked in water for different periods. (b) The corresponding shear adhesion strength of the PAE_0/T hydrogel after being soaked in water for different periods.

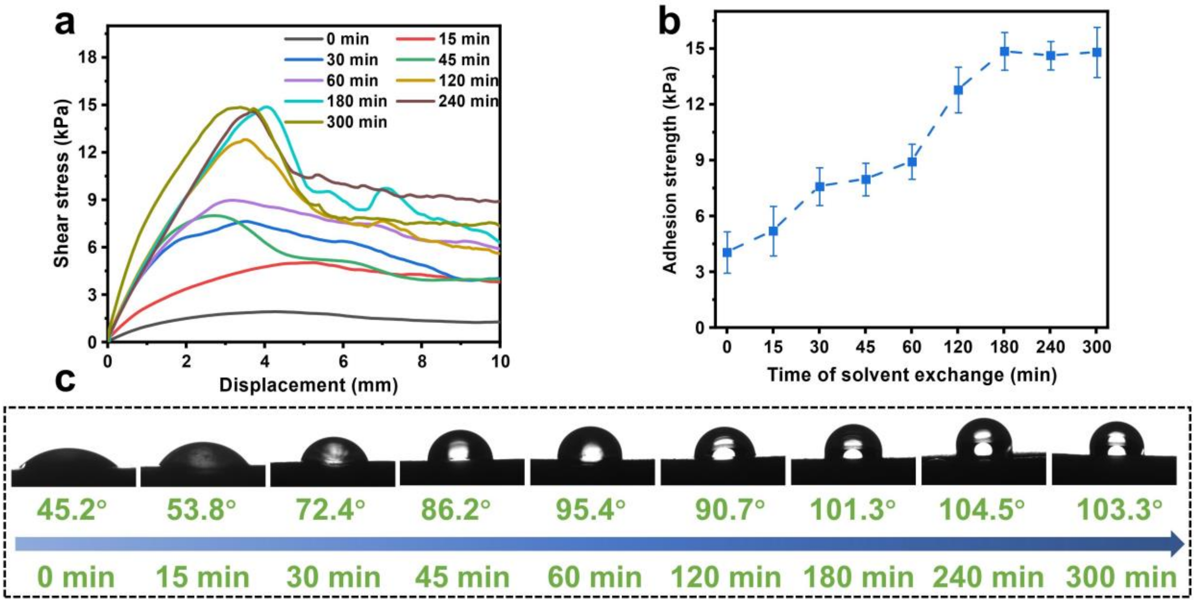

Figure S27. Adhesion performance of the $PAE_{1/3}$/T hydrogel underwater with different solvent exchange times. (a) Shear adhesion stress *versus* displacement of the $PAE_{1/3}$/T hydrogel underwater with different solvent exchange times. (b) Summary of adhesion strength of the $PAE_{1/3}$/T hydrogel underwater with different solvent exchange times. (c)WCA of the $PAE_{1/3}$/T hydrogel underwater with different solvent exchange times.

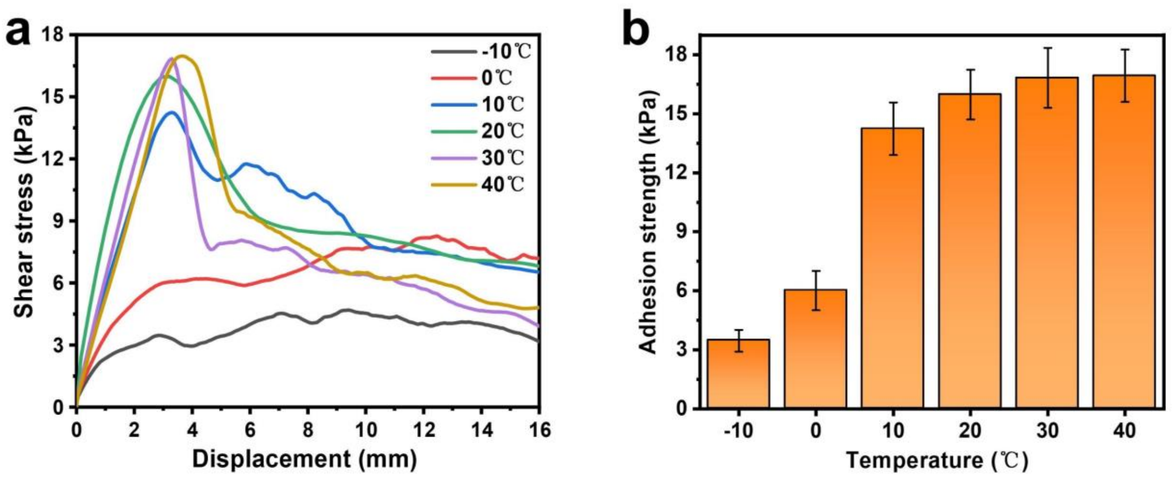

Figure S28. Adhesion performance of the $PAE_{1/3}$/T hydrogel underwater at varying water temperatures. (a) Shear adhesion stress *versus* displacement of the $PAE_{1/3}$/T hydrogel underwater at different water temperatures. (b) Summarized adhesion strength of the $PAE_{1/3}$/T hydrogel underwater at different water temperatures.

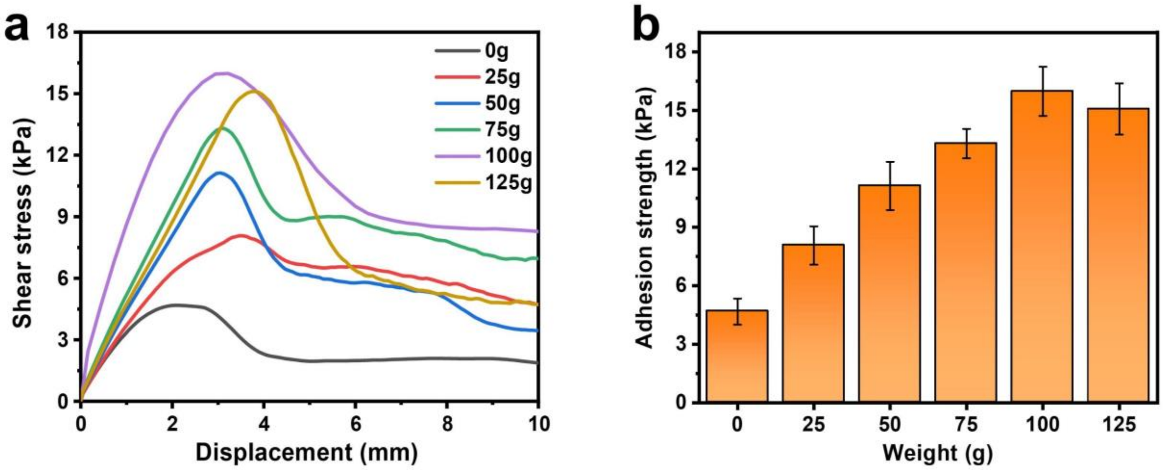

Figure S29. Underwater adhesion performance of the $PAE_{1/3}$/T hydrogel under varying preloads. (a) Shear adhesion stress *versus* displacement for the $PAE_{1/3}$/T hydrogel underwater, with different preloads applied. (b) Summarized adhesion strength results for the $PAE_{1/3}$/T hydrogel underwater with different preload applied.

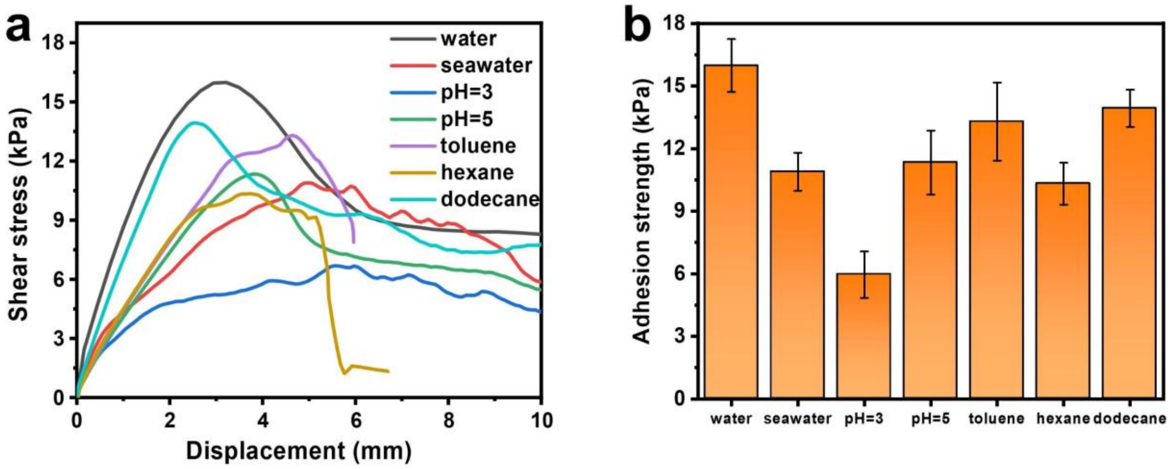

Figure 30. Adhesion performance of the PAE$_{1/3}$/T hydrogel in different solvents. (a) Shear adhesion stress *versus* displacement the PAE$_{1/3}$/T hydrogel in different solvents. (b) Summarized adhesion strength of the PAE$_{1/3}$/T hydrogel in different solvents.

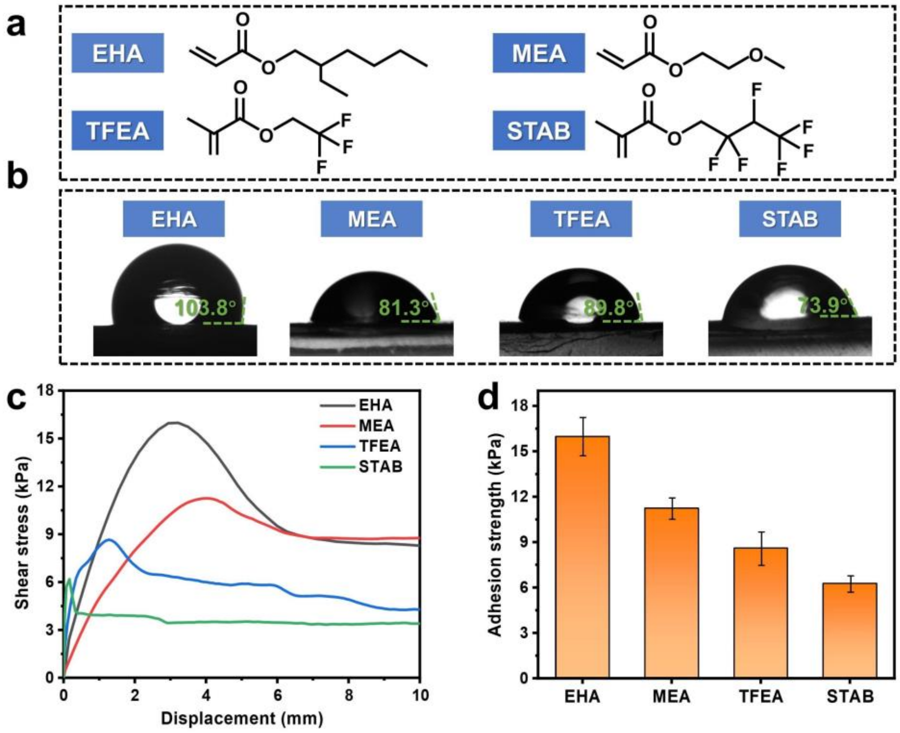

Figure S31. Adhesion performance of the PS hydrogel prepared with various hydrophobic monomers. (a) Chemical structures of the hydrophobic monomers EHA, MEA, TFEA, and STAB. (b) Effect of the hydrophobic monomers on the WCA of PS hydrogels. (c) Shear adhesion stress *versus* displacement for the PS hydrogel prepared with different hydrophobic monomers. (d) Summary of adhesion strength for the PS hydrogel prepared with different hydrophobic monomers.

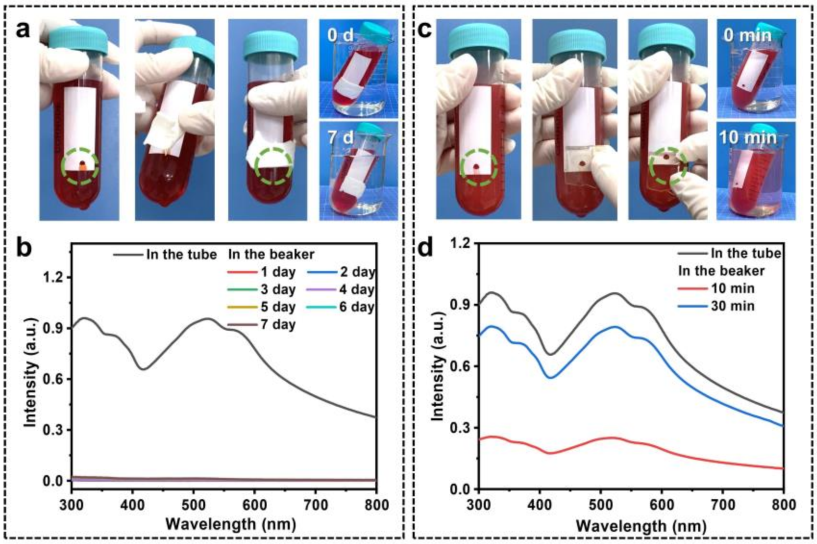

Figure S32. Application of the PS hydrogel as an instant sealing tape. (a) Photographs showing that the $PAE_{1/3}$/T gel can cease the leakage of a perforated centrifuge tube. The sealing is reliable underwater for at least 7 days. (b) UV-Vis spectra of water in the beaker at different time periods and solution in the tube, respectively. (c) Photographs showing that the PAE_0/T gel fails to prevent the leakage of a perforated tube. (d) UV-Vis spectra of water in the beaker and solution in the tube, respectively.

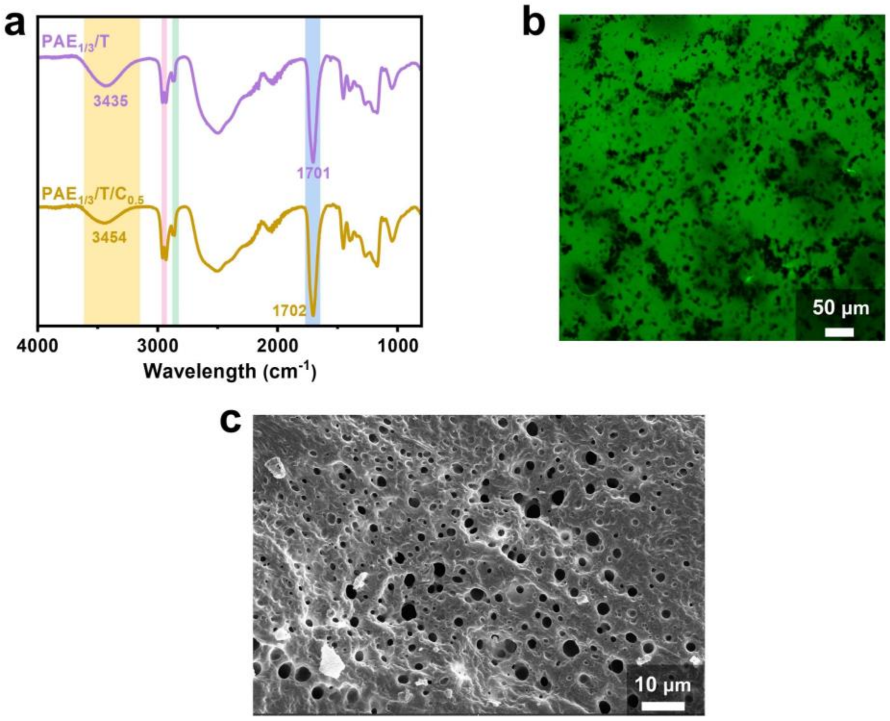

Figure S33. Characterization on the MWCNT-COOH-containing PS hydrogel. (a) ATR FT-IR spectra of the $PAE_{1/3}$/T gel and the $PAE_{1/3}/T/C_{0.5}$ gel, respectively. (b) CLSM image of the $PAE_{1/3}/T/C_{0.5}$ gel. (c) SEM image of the $PAE_{1/3}/T/C_{0.5}$ gel.

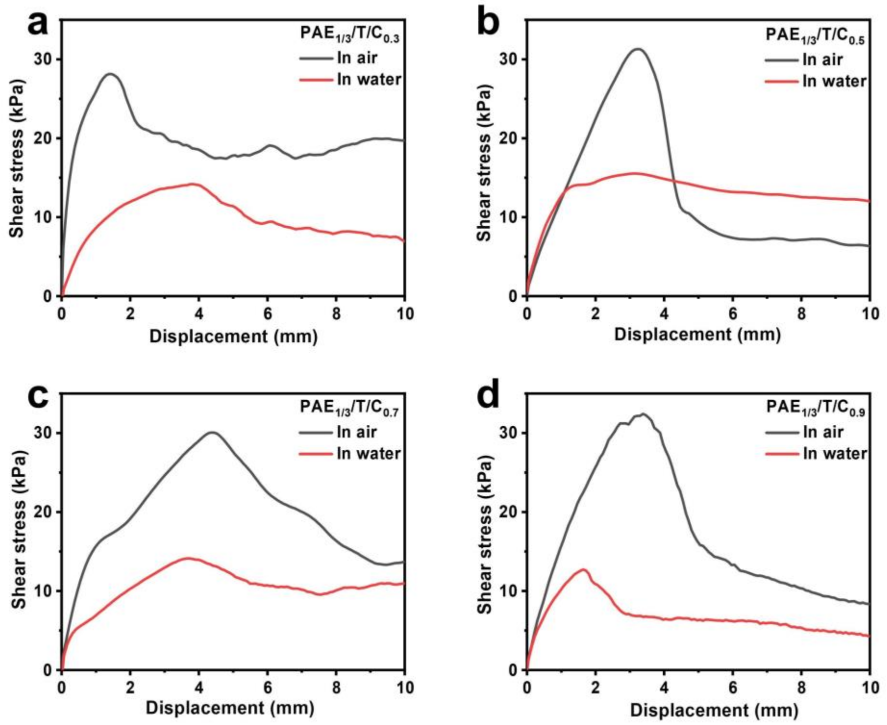

Figure S34. Adhesion capability of $PAE_{1/3}/T/C_z$ hydrogels with different MWCNT contents to glass in air and water. (a) Shear adhesion stress *versus* displacement of the $PAE_{1/3}/T/C_{0.3}$ gel in air and in water, respectively. (b) Shear adhesion stress *versus* displacement of the $PAE_{1/3}/T/C_{0.5}$ gel in air and in water, respectively. (c) Shear adhesion stress *versus* displacement of the $PAE_{1/3}/T/C_{0.7}$ gel in air and in water, respectively. (d) Shear adhesion stress *versus* displacement of the $PAE_{1/3}/T/C_{0.9}$ gel in air and in water, respectively.

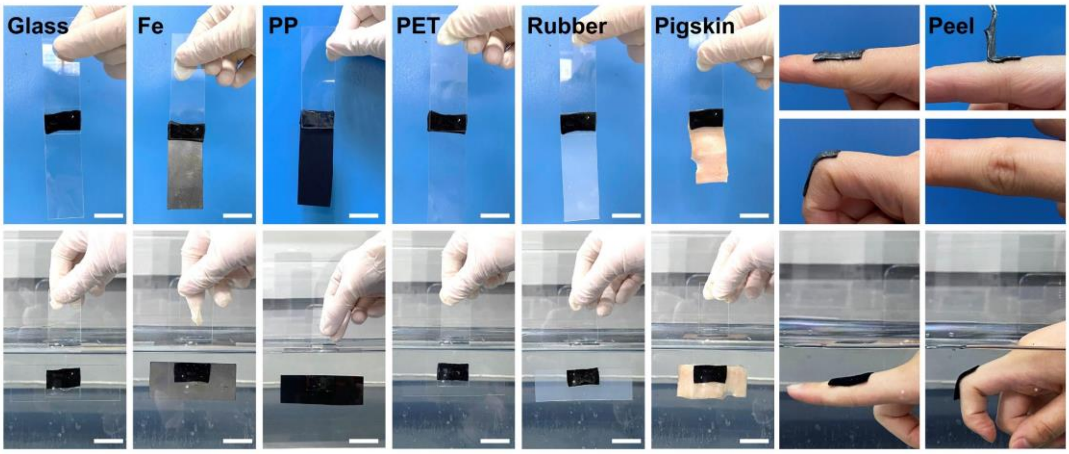

Figure S35. Photographs showing the universal adhesion of the $PAE_{1/3}/T/C_{0.5}$ hydrogel to different substrates in air and water, respectively. The scale bars represent 2 cm.

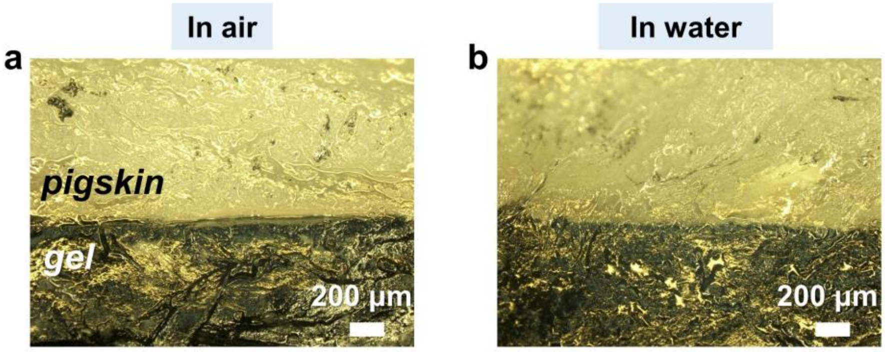

Figure S36. (a) The microscope image of the gel-skin interface in air. (b) The microscope image of the gel-skin interface in water.

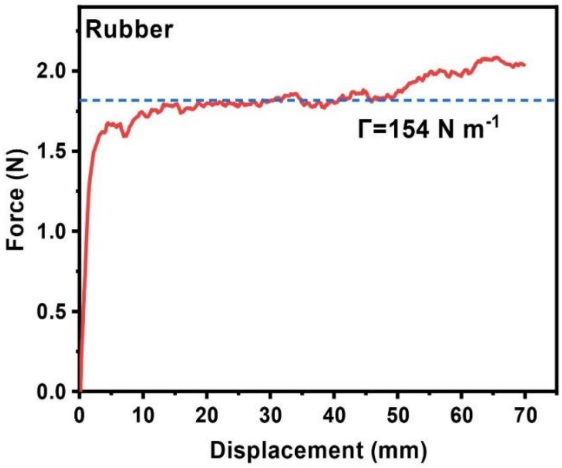

Figure S37. Curve of the peeling force *versus* displacement of the $PAE_{1/3}/T/C_{0.5}$ gel on rubber.

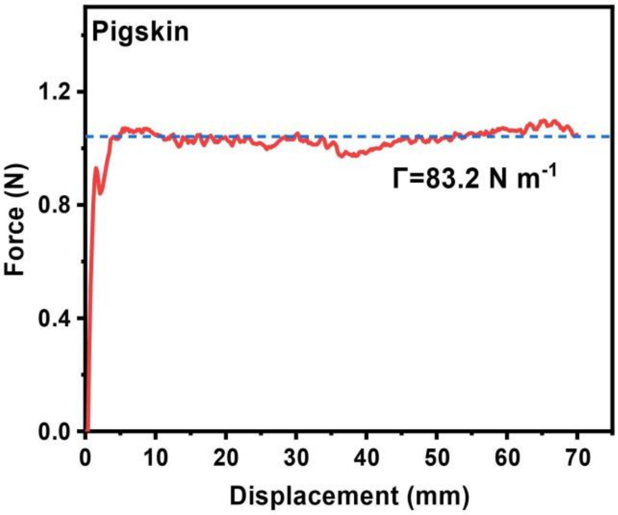

Figure S38. Curve of the peeling force *versus* displacement of the $PAE_{1/3}/T/C_{0.5}$ gel on pigskin.

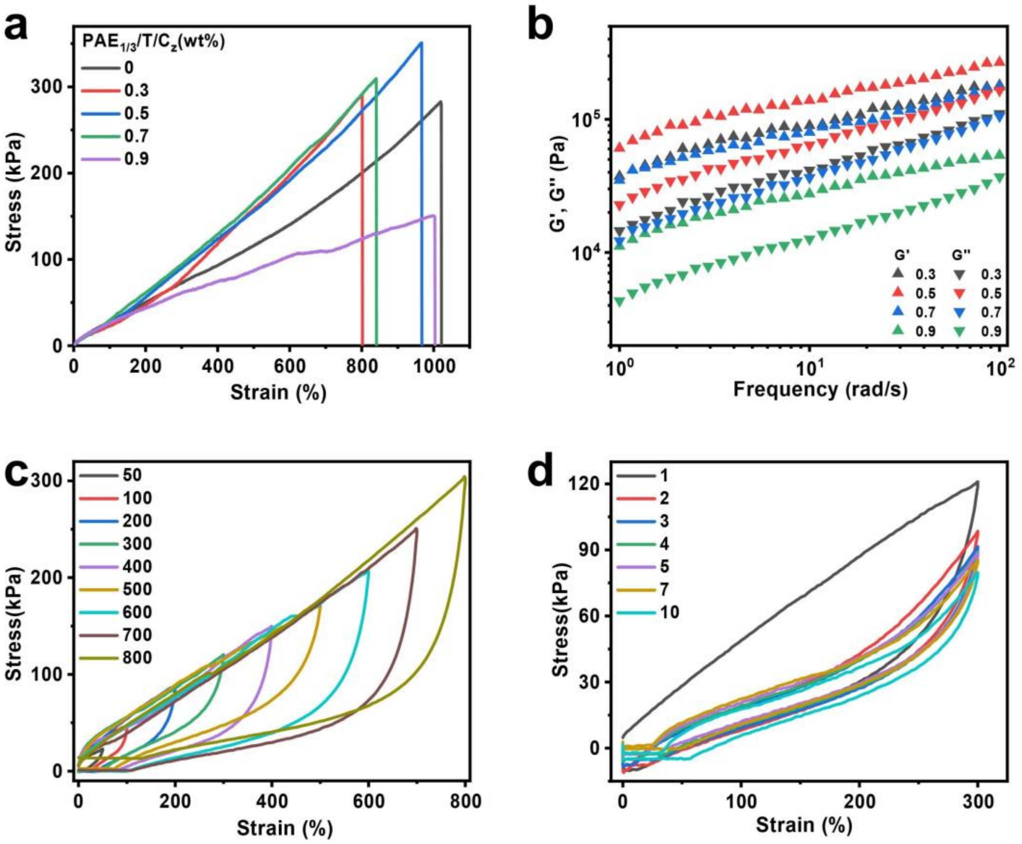

Figure S39. Influence of MWCNT-COOH content on the mechanical properties of the PAE/T/C hydrogel. (a) Tensile stress-strain curves of $PAE_{1/3}/T/C_z$ hydrogels with different MWCNT-COOH contents. (b) Storage and loss modulus of all $PAE_{1/3}/T/C_z$ gels in a frequency range of 1 to 100 rad s^{-1}. (c) Tensile loading-unloading curves of the $PAE_{1/3}/T/C_{0.5}$ gel at different strains. (d) Cyclic tensile loading-unloading curves of the $PAE_{1/3}/T/C_{0.5}$ gel at a fixed strain of 300% with no resting time.

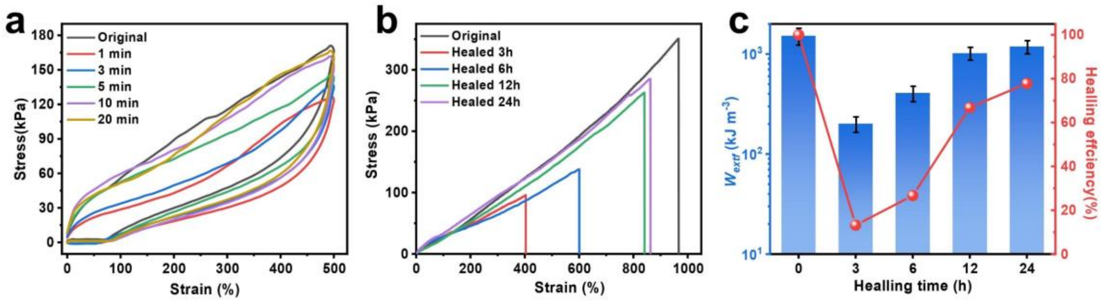

Figure S40. Self-recovery and self-healing capability of the $PAE_{1/3}/T/C_{0.5}$ hydrogel. (a) Tensile loading-unloading stress-strain curves of the $PAE_{1/3}/T/C_{0.5}$ gel at a fixed strain of 500% with different recovery times. (b) Tensile stress-strain curves of the $PAE_{1/3}/T/C_{0.5}$ gel with different healing times. (c) Work of extension at fracture (W_{extf}) and healing efficiency of the $PAE_{1/3}/T/C_{0.5}$ gel with different healing times.

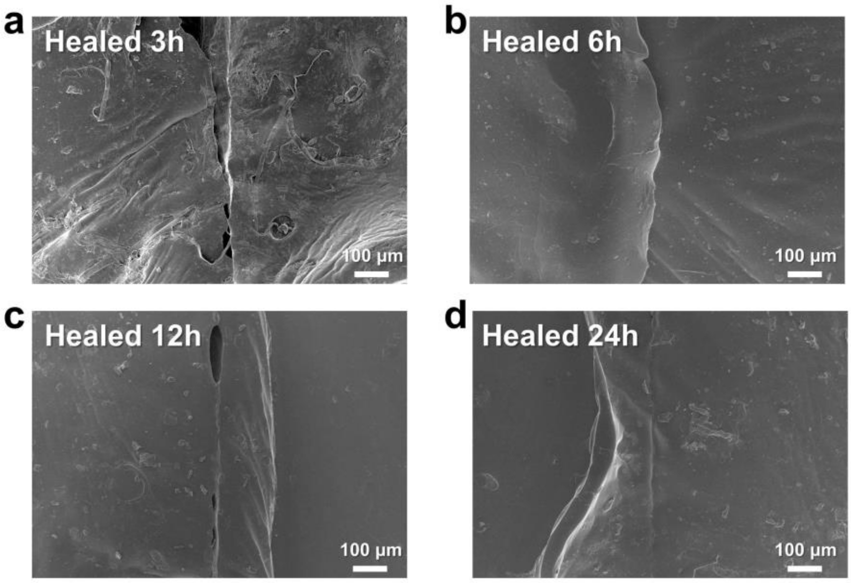

Figure S41. Self-healing process of the PAE$_{1/3}$/T/C$_{0.5}$ gel observed by SEM. (a) SEM image of the gel after being healed for 3 h. (b) SEM image of the gel after being healed for 6 h. (c) SEM image of the gel after being healed for 12 h. (d) SEM image of the gel after being healed for 24 h.

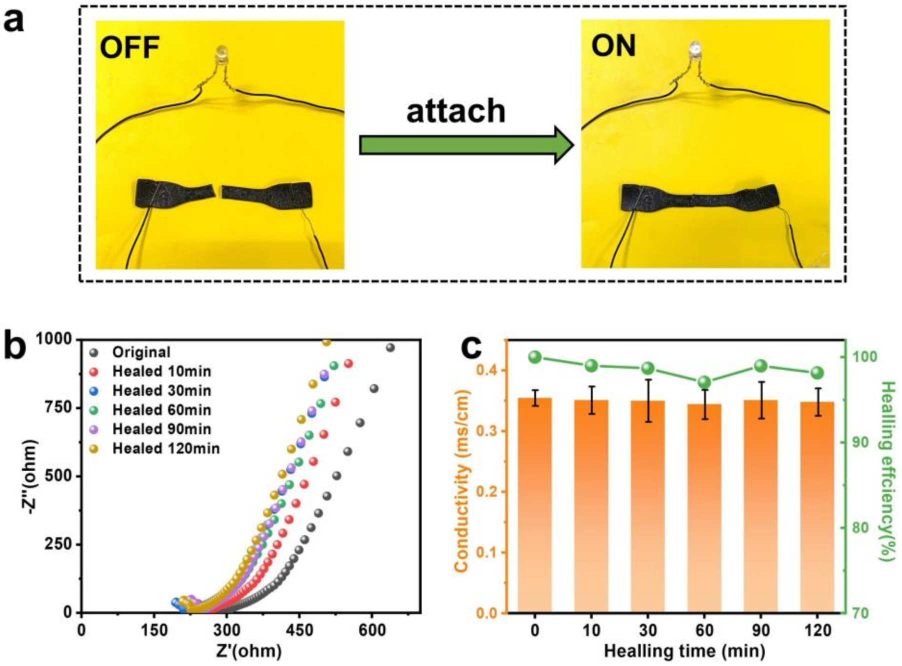

Figure S42. Effect of self-healing time on the conductivity of the PAE$_{1/3}$/T/C$_{0.5}$ hydrogel. (a) Photographs showing that two pieces of the PAE$_{1/3}$/T/C$_{0.5}$ gel can self-heal into an intact one to light up an LED bulb. (b) EIS curves of the PAE$_{1/3}$/T/C$_{0.5}$ gel with different healing times. (c) The conductivity of the PAE$_{1/3}$/T/C$_{0.5}$ gel with different healing times and corresponding healing efficiency defined by the conductivity ratio of the healed gel to the original one.

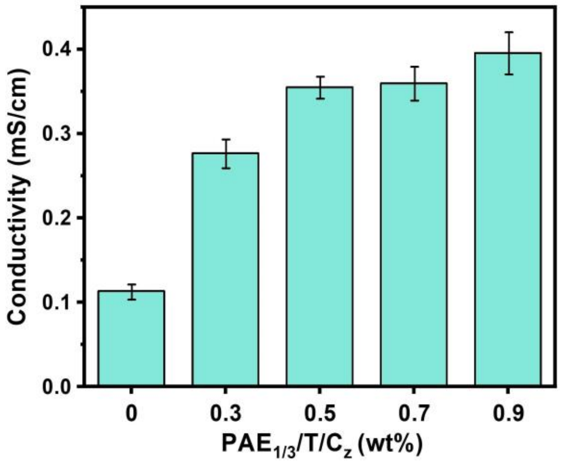

Figure S43. Effect of MWCNT-COOH content on the conductivity of $PAE_{1/3}/T/C_z$ hydrogels.

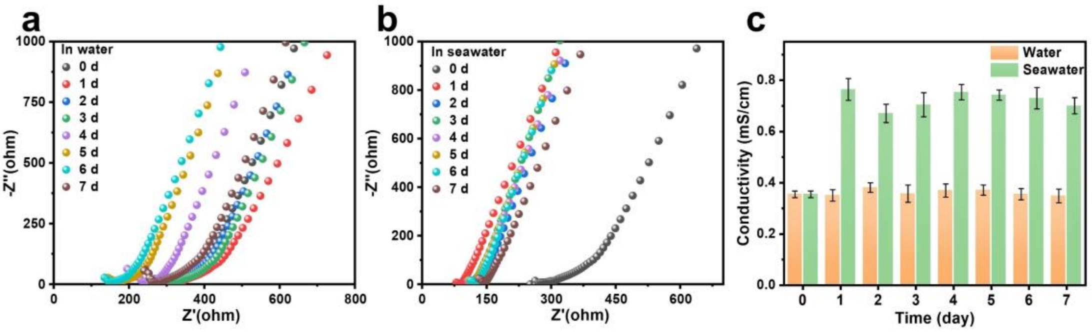

Figure S44. Influence of soaking time in aqueous solutions on the conductivity of the $PAE_{1/3}/T/C_{0.5}$ hydrogel. (a) EIS curves of the $PAE_{1/3}/T/C_{0.5}$ gel with different soaking time periods in water. (b) EIS curve of the $PAE_{1/3}/T/C_{0.5}$ gel with different soaking time periods in artificial seawater. (c) Corresponding conductivity of the $PAE_{1/3}/T/C_{0.5}$ gel with different soaking time periods in water and artificial seawater.

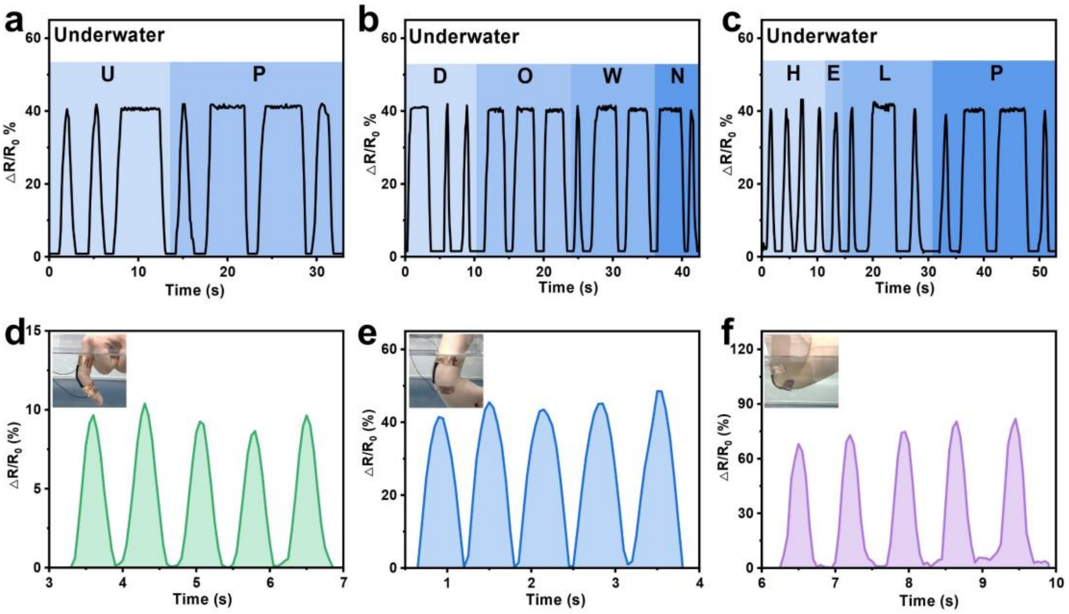

Figure S45. Underwater communication by using the PAE$_{1/3}$/T/C$_{0.5}$ hydrogel as the sensor module in the electronics. (a, b, c) The raw signals of different Morse codes represented by the fast-mode and slow-mode relative resistance change of the hydrogel. (d, e, f) Detection of human body movement underwater by using the hydrogel as a sensor.

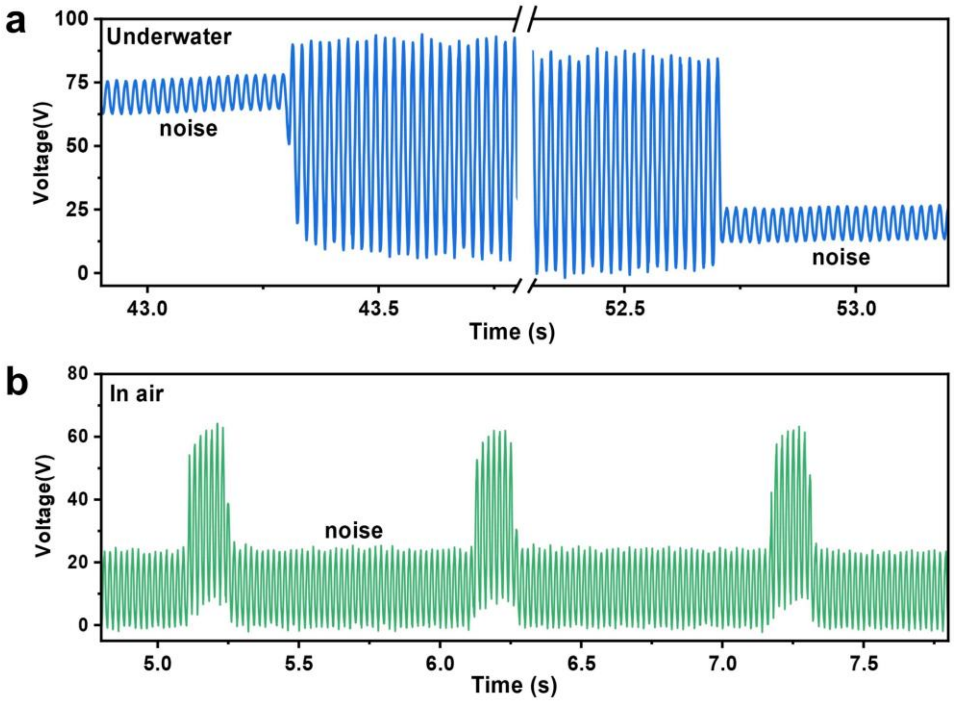

Figure S46. (a) The output noise signal and triboelectric signal when the rubber glove separated from and contacted the water surface, respectively. (b) The output noise signal and triboelectric signal when the rubber glove separated from and contacted the TENG surface in air, respectively.

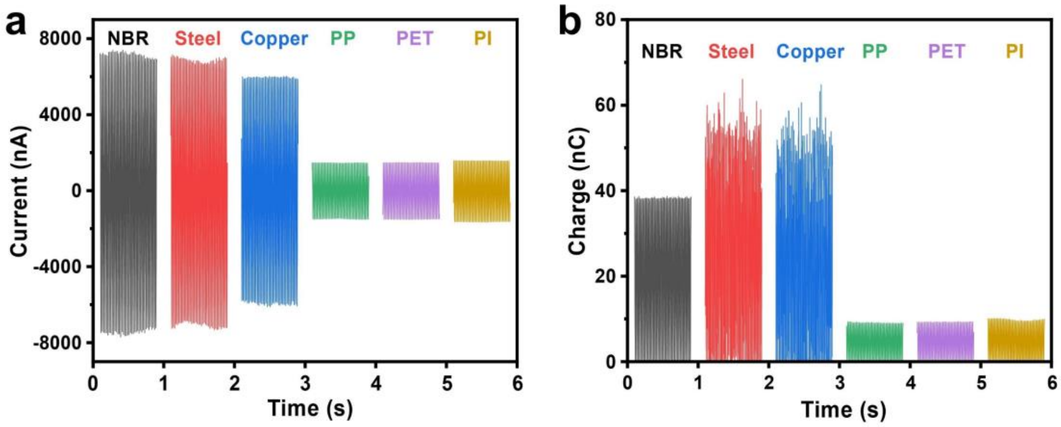

Figure S47. The output performance of hydrogel-based TENG underwater by touching the water surface with different materials. (a) The short-circuit current. (b) The charge transfer amount.

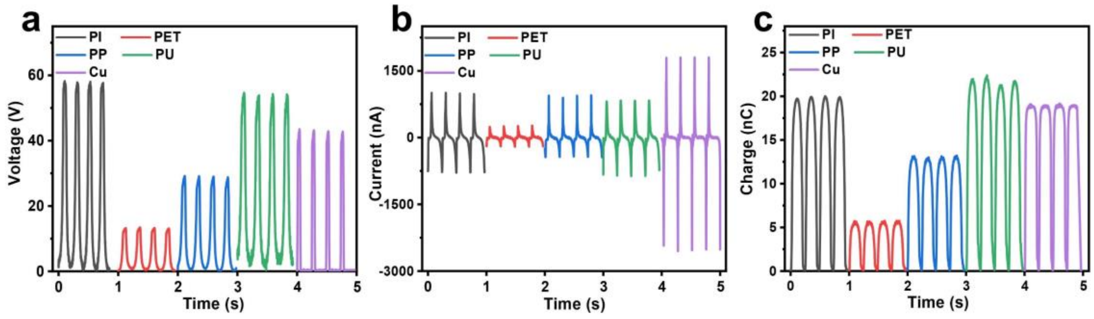

Figure S48. Influence of triboelectric material type on the output performance of hydrogel-based TENG an operating frequency of 3 Hz in air. (a) The open-circuit voltage. (b) The short-circuit current. (c) The charge transfer amount.

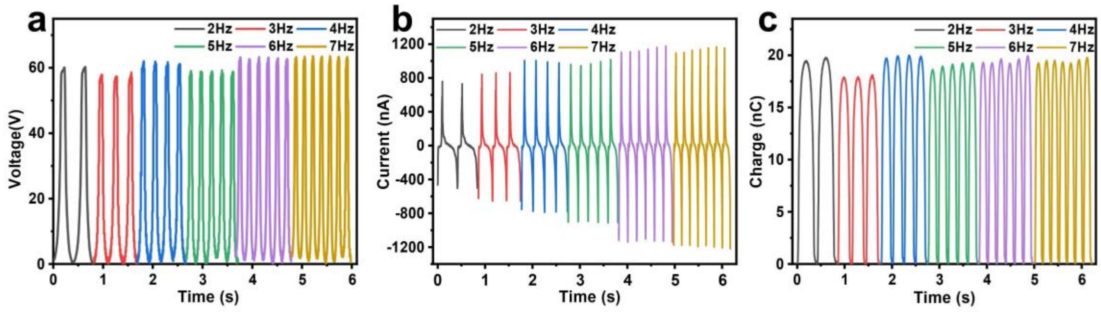

Figure S49. The influence of frequency range on the output performance hydrogel-based TENG in air. (a) The open-circuit voltage. (b) The short-circuit current. (c) The charge transfer amount.

Supplementary Table S1. Comparison of main components and total cost of state-of-the-art ionogels and PS hydrogel in this work.

Reference	Main components and unit price[a]	Total cost
Adv. Mater. 2021, 33, 2008479	Methacryloxyethyltrimethyl ammonium chloride (MATAC, 0.82 \$ g^{-1})	3.36 \$ g^{-1}
	2.2,2-Diethoxyacetophenone, bis(trifluoromethane)sulfonimide lithium salt (LiTFSI, 5.96 \$ g^{-1})	
	Butyltrimethylammonium chloride ([N4111] Cl, 0.16 \$ g^{-1})	
Adv. Mater. 2021, 33, 2105306	2,2,2-Trifluoroethyl acrylate (TFEA, 5.34 \$ g^{-1})	4.61 \$ g^{-1}
	1-Ethyl-3-methylimidazolium bis(trifluoromethylsulfonyl)imide ([EMIM][TFSI], 4.66 \$ g^{-1})	
	Acrylamide (AAm, 0.044 \$$g^{-1}$)	
This work	Acrylic acid (AAc, 0.242 \$ g^{-1})	0.058 \$ g^{-1}
	2-ethylhexyl acrylate (EHA, 0.044 \$ g^{-1})	
	tannic acid (TA, 0.791 \$ g^{-1})	
	Dimethyl sulfoxide (DMSO, 0.034 \$ g^{-1})	
	Deionized water (DI, 0.0016 \$ g^{-1})	

[a]All unit prices are sourced from Aladdin Biochemical Technology Co., Ltd (Shanghai, China), and are denominated in U.S. dollar.

Supplementary Table S2: Composition contents of all hydrogels in this work.

	**AAc**	**EHA**	**TA**	**KPS**	**MBAA**	**H_2O**
	(mol)	(mol)	(mg)[a]	(mol/mol)[b]	(mol/mol)[c]	(g)
**PAE$_{0}$/T**	0.03	0	20	0.2	0.1	10
	**AAc**	**EHA**	**TA**	**KPS**	**MWCNT-COOH**	**DMSO**
	(mol)	(mol)	(mg)[a]	(mol/mol)[b]	(mg)[d]	(g)
**PAE$_{1/6}$/T**	0.03	0.005	20	0.2	0	10
**PAE$_{1/3}$/T**	0.03	0.01	20	0.2	0	10
**PAE$_{2/3}$/T**	0.03	0.02	20	0.2	0	10
**PAE$_{1}$/T**	0.03	0.03	20	0.2	0	10
**PAE$_{1/3}$/T$_{0}$**	0.03	0.01	0	0.2	0	10
**PAE$_{1/3}$/T$_{10}$**	0.03	0.01	10	0.2	0	10
**PAE$_{1/3}$/T$_{30}$**	0.03	0.01	30	0.2	0	10
**PAE$_{1/3}$/T/C$_{0.3}$**	0.03	0.01	20	0.2	42	10

**$PAE_{1/3}/T/C_{0.5}$**	0.03	0.01	20	0.2	70	10
**$PAE_{1/3}/T/C_{0.7}$**	0.03	0.01	20	0.2	98	10
**$PAE_{1/3}/T/C_{0.9}$**	0.03	0.01	20	0.2	126	10
**PA_4E_1/T**	0.04	0.01	20	0.2	70	10
**PA_5E_1/T**	0.05	0.01	20	0.2	70	10
**PA_6E_1/T**	0.06	0.01	20	0.2	70	10
	**AAc**	**MEA**	**TA**	**KPS**	**MBAA**	**DMSO**
	(mol)	(mol)	(mg)[a]	(mol/mol)[b]	(mol/mol)[c]	(g)
**$PAM_{1/3}/T$**	0.03	0.01	20	0.2	0	10
	**AAc**	**TFEMA**	**TA**	**KPS**	**MBAA**	**DMSO**
	(mol)	(mol)	(mg)[a]	(mol/mol)[b]	(mol/mol)[c]	(g)
**$PAF_{1/3}/T$**	0.03	0.01	20	0.2	0.1	10
	**AAc**	**MEA**	**TA**	**KPS**	**MBAA**	**DMSO**
	(mol)	(mol)	(mg)[a]	(mol/mol)[b]	(mol/mol)[c]	(g)
**$PAS_{1/3}/T$**	0.03	0.01	20	0.2	0.1	10

[a] In 10 g of the precursor solution.

[b] Relative to the sum of AAc and EHA monomers.

[c] Relative to AAc monomer.

[d] Relative to the total mass of the precursor solution.

Supplementary Movie Captions

Movie S1: Contrasting Underwater Adhesion Behaviors of the PS $PAE_{1/3}/T$ gel and the NPS PAE_0/T gel

Movie S2: Demonstration of the PS hydrogel as an instant glue to pick up substrates in water

Movie S3: Contrasting Adhesion Behaviors in Air of the PS $PAE_{1/3}/T$ gel and the NPS PAE_0/T gel

Movie S4: Operation of a Hydrogel-Based TENG Underwater

Movie S5: Operation of a Hydrogel-Based TENG in Air

Supplementary Reference

[1] K. Chen, Z. Wu, Y. Liu, Y. Yuan, C. Liu, *Adv. Funct. Mater.* **2022**, 32, 2109687.
